# Evaluating Thiram‐Induced Embryotoxicity Using Integrated In Silico, In Vitro, and Transcriptomic Approaches

**DOI:** 10.1002/tox.70103

**Published:** 2026-04-21

**Authors:** Chia‐Chi Ho, Chun‐Wei Tung, B. Linju Yen, Chen‐Yi Weng, Ju‐Hsin Hsu, Ming‐Hsien Tsai, Salim Arrokhman, Pinpin Lin

**Affiliations:** ^1^ National Institute of Environmental Health Sciences National Health Research Institutes Zhunan Taiwan; ^2^ Institute of Biotechnology and Pharmaceutical Research National Health Research Institutes Zhunan Taiwan; ^3^ Institute of Cellular and System Medicine National Health Research Institutes Zhunan Taiwan; ^4^ Center for Biomedical Research National Research and Innovation Agency (BRIN) Cibinong Bogor Indonesia

**Keywords:** embryotoxicity, human pluripotent embryonal carcinoma cells (NT2), mouse embryonic stem cells (mESCs), neural crest differentiation, thiram

## Abstract

Long‐term exposure to low‐dose food contact materials (FCMs) has raised concerns regarding developmental toxicity. In the present study, we prioritized FCMs with potential developmental toxicity using a weight‐of‐evidence computational model, which predicted 127 chemicals to be of high concern. From these, we selected 7 chemicals—representing both high and low concern categories—and evaluated their potential embryotoxicity using the mouse embryonic stem cell test (mEST). Among the selected chemicals, thiram most strongly inhibited cardiac differentiation in mEST. We further examined the effects of thiram on morphology and expression of differentiation‐related genes in mouse embryonic stem cells (mESCs). Treatment with thiram inhibited 50% early differentiation in mESCs, suppressed the expression of markers associated with the three‐germ layers, but increased the expression of neurectoderm markers during early embryogenesis. Additionally, treatment with 20 ng/mL thiram, which was the lowest‐observed‐effect concentration of cytotoxicity, disrupted neuronal differentiation in both mESCs and human pluripotent embryonal carcinoma NT2 cells. Finally, based on transcriptome analysis, 20 and 30 ng/mL thiram disrupted the neural crest differentiation pathway, altering the expression of genes including *homeobox A1 (HOXA1), homeobox B1 (HOXB1), Heart And Neural Crest Derivatives Expressed 1 (HAND1)*, *Distal‐Less Homeobox 5 (DLX5)*, and *transcription factor AP‐2 alpha (TFAP2A)* in NT2 cells. Therefore, disruption of neural crest differentiation is one of the potential mechanisms underlying thiram‐induced embryotoxicity. The integrated alternative approach adopted in the present study to identify mechanism‐based biomarkers for thiram‐induced embryotoxicity in a human‐relevant model could facilitate safety assessment for data‐poor chemicals in future.

## 1 Introduction

Food contact materials (FCMs) are a group of chemicals used in the production of food storage materials such as paper and board, plastics, metals, glass, adhesives, and printing inks. FCMs play a pivotal role in modern society by protecting food from contamination and thereby reducing food waste [1]. However, thousands of chemicals in paper and board packaging alone may migrate into food [2]. Moreover, most FCMs encountered in daily life lack comprehensive toxicological data. Therefore, there is an urgent need to develop efficient strategies to prioritize chemicals for further toxicological evaluation [3, 4]. Traditional animal studies are costly, time‐consuming, and often fail to accurately predict human responses [5]. At the start of the 21st century, the National Research Council in the United Sates envisioned a shift in toxicity testing toward high‐throughput in vitro and computational approaches based on human biology to assess potential adverse effects of chemical exposure [6]. Toxicity pathways, defined as cellular mechanisms whose disruption can lead to adverse outcomes, represent the mode of action concept and are essential for developing mechanism‐based safety assessments [7]. Building on such a framework, initiatives such as Safety Evaluation Ultimately Replacing Animal Testing in Europe and New Approach Methodologies are advancing chemical safety evaluation globally without animal testing [7, 8]. To evaluate chemical safety without relying on vertebrate models, a tiered framework has been proposed that incorporates new methodologies, including in silico, in vitro, and non‐mammalian in vivo models [8, 9, 10, 11]. This integrated approach may enhance the confidence of safety assessments by characterizing the mode of action or mechanisms underlying chemical‐induced adverse effects, or by conducting evaluations in human‐relevant models.

In the present study, we followed a tiered framework to assess the potential early embryotoxicity of FCMs (Figure 1). First, we applied a weight‐of‐evidence (WoE) model to identify FCMs with potential reproductive and developmental toxicity [12]. Next, we conducted dose–response studies to validate the embryotoxic effects of selected FCMs using the mouse embryonic stem cell test (mEST). As a result, thiram was predicted to be of high embryotoxicity concern. Thiram is used as an accelerator and vulcanizing agent in rubber production, as well as an activator in plastics manufacturing [13]. It is listed as a food contact substance and is used as an adhesive in reusable rubber products [14]. Thiram is also the most widely used dithiocarbamate (DTC) fungicide, protecting seeds, fruits, vegetables, ornamental plants, and turf from various fungal diseases, and preventing post‐harvest deterioration during storage and transport [15, 16]. Globally, the annual use of DTC fungicides is estimated to be 25 000–35 000 tons [17]. In the United States, the estimated average annual usage of thiram from 1998 to 2018 was 140 tons, while South Korea imported approximately 150 tons between 2013 and 2016 [18, 19]. In addition, approximately 631 000 pounds of thiram are used each year for the treatment of around 1.3 billion pounds of seeds in the USA [20]. These findings indicate that humans are likely exposed to thiram through diet and environmental sources, raising potential health concerns.

**FIGURE 1:**
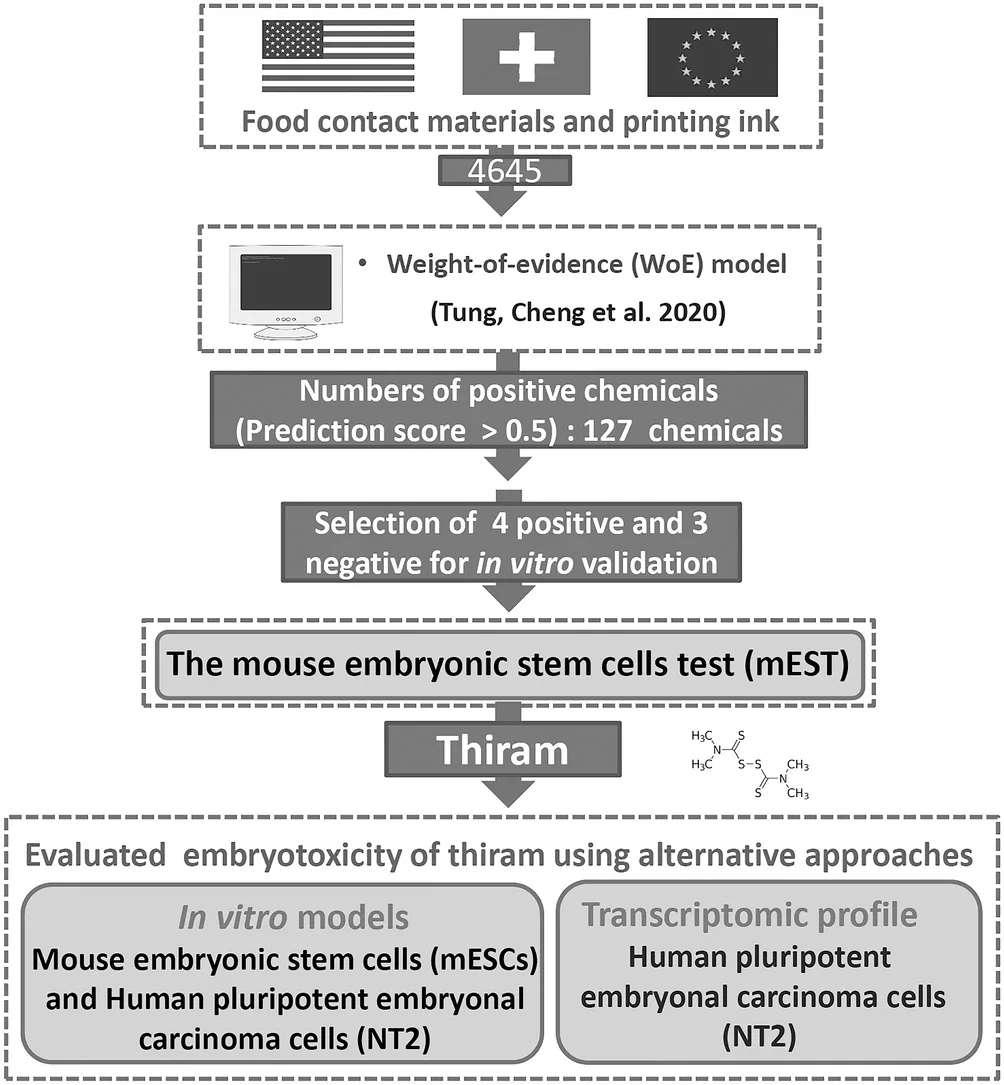
Workflow for assessing the potential embryonic toxicity of thiram.

Thiram has been associated with multiple toxic effects, including thyroid disruption [21], as well as developmental, hepatic, and neurotoxicity in animals [15, 22, 23]. Although several studies have reported developmental effects of thiram in animals, the underlying toxicological mechanisms remain poorly understood. Advances in molecular biology, biotechnology, and bioinformatics in the 21st century now enable the investigation of chemical‐induced adverse outcomes through elucidation of molecular mechanisms, or modes of action, which can improve chemical safety evaluation and risk assessment [7, 24, 25]. Therefore, understanding the toxicological mechanisms of thiram during embryonic development is of critical importance. In the present study, we employed a combination of alternative approaches to elucidate the embryotoxicity mechanisms of thiram, including in silico, mouse and human in vitro models [26], and a transcriptomics‐based approach [27] (Figure 1). Our findings suggest that disruption of neural crest differentiation may be a key toxicological target underlying thiram‐induced embryotoxicity. The present study demonstrated that in silico approaches effectively predicted the developmental toxicity of FCMs, which was then confirmed using mouse and human in vitro models. This work could facilitate the establishment of an integrated strategy that combines in silico prediction with targeted in vitro validation.

## 2 Materials and Methods

### 2.1 Chemicals

Thiram, fluorescein, eosin Y, 2,2′‐methylenebis(4‐methyl‐6‐tert‐butylphenol) (2 Mb), 2‐ethyl‐4‐methylimidazole (2E4M), dodecylbenzene (DB), 1,2,3,6‐tetrahydrophthalic anhydride (TDA), dimethyl sulfoxide (DMSO), poly‐d‐lysine (PDL), cytosine β‐D‐arabinofuranoside (AraC), and uridine were obtained from Sigma‐Aldrich (St. Louis, MO, USA). Laminin (LAM) was purchased from Invitrogen (Hayward, CA, USA).

### 2.2 In Silico Prediction

We initially collected 4645 chemicals from three regulatory and industry‐related sources: (1) the U.S. Food and Drug Administration (FDA) list of indirect additives used in food contact substances [28]; (2) the European Union (EU) plastics regulation list [29]; and (3) the Swiss packaging ink list [30]. These chemicals were then prioritized for potential reproductive and developmental toxicities using a WoE computational model [12], which incorporated three predictive tools: (1) the OECD Quantitative Structure–Activity Relationship (QSAR) Toolbox, utilizing the Developmental and Reproductive Toxicity (DART) model [31]; (2) ChemDIS [32], which integrates Gene Ontology [33] and Disease Ontology [34]; and (3) Derek Nexus [35], using a reproductive toxicity prediction model.

### 2.3 Mouse Embryonic Stem Cells (mESCs), BALB/c 3T3 Cells and Human Embryonal Carcinoma NT2 Cells

The mouse embryonic stem cell line ES‐D3 [D3] (ATCC CRL‐1934; RRID: CVCL_4378), the fibroblast cell line BALB/3T3 clone A31 (ATCC CCL‐163; RRID: CVCL_0184), and the human pluripotent embryonal carcinoma cell line NTERA‐2 cl.D1 [NT2/D1] (ATCC CRL‐1973; RRID: CVCL_3407) were obtained from the American Type Culture Collection (ATCC; Manassas, VA, USA). All cell lines were expanded and cryopreserved immediately upon receipt, and aliquots from early passages were used for all experiments. None are listed as misidentified or contaminated in the ICLAC register. ES‐D3, BALB/3T3 and NTERA‐2 cl.D1 were authenticated through short‐tandem repeat profiling, and they were all negative for mycoplasma contamination.

The mESCs (D3; *Mus musculus* , male embryo) were cultured under conditions that maintained their undifferentiated state, following the protocol published in Nature Protocol [36]. Cells were used before passage 24, and they were maintained on mitotically inactivated mouse embryonic fibroblast (MEF) feeder layers ( *Mus musculus* , embryonic fibroblast, RRID:CVCL_5251; ATCC SCRC‐1040, Manassas, Virginia, USA). The culture medium consisted of Mouse ES Cell Basal Medium (ATCC SCRR‐2011, Manassas, Virginia, USA), supplemented with 15% fetal bovine serum (FBS; Hyclone, Marlborough, MA, USA), 0.1 mM 2‐mercaptoethanol, and 1,000 U/mL mouse leukemia inhibitory factor (LIF; Millipore, Darmstadt, Germany). The BALB/c 3T3 cells ( *Mus musculus* , embryo) were cultured in Dulbecco's Modified Eagle Medium (DMEM; ATCC, Catalog No. 30‐2002, Manassas, Virginia, USA) supplemented with 10% bovine calf serum (Cytiva, Marlborough, MA, USA), and were used before passage 20. The human embryonal carcinoma cells (NT2, *Homo sapiens* , male testicular embryonal carcinoma) were maintained in DMEM (Gibco, CA, USA) supplemented with 4 mM L‐glutamine, 3.7 g/L sodium bicarbonate, and 10% fetal bovine serum (FBS; Gibco, CA, USA), and were used before passage 40. All cell lines were incubated at 37°C in a humidified atmosphere of 5% CO_2_ and 95% air. Cells were expanded and cryopreserved immediately upon receipt, with early‐passage aliquots used for all experiments, and they were maintained at 70%–80% confluence. Experiments were performed with three independent biological replicates, each with two or three technical replicates to ensure reproducibility.

### 2.4 Mouse Cardiomyocyte Differentiation

We differentiated mESCs into cardiomyocytes following the protocol described [36]. In the absence of LIF, mESCs underwent spontaneous differentiation. On the underside of a 100‐mm Petri dish lid, 20‐μL drops of mESC suspension (3.75 × 10^4^ cells/mL) were placed. The lid was then inverted, allowing the cells to be cultured in hanging drops for 3 days. After this period, the resulting aggregates (embryoid bodies, EBs) [36] were transferred into 96‐well Ultra‐Low Attachment plates (Costar, New York, NY, USA). Two days later, EBs were transferred individually to 24‐well plates, where they continued to develop into various embryonic tissues. Differentiation into contracting cardiomyocytes, indicated by cell beating, was observed under light microscopy on the 5th day after plating in 24‐well plates. The percentage of differentiation was calculated as the ratio of wells containing beating cells in chemically treated plates to those in 0.1% DMSO‐treated control plates. In the present experiments, beating cells were observed in all 24 wells of the 0.1% DMSO‐treated plates. The inhibition of differentiation (ID_50_) was determined from dose–response curves as the concentration at which 50% of wells lacked beating cardiomyocytes. The ID_50_ value was calculated using GraphPad Prism 5 software (GraphPad Software, Boston, MA, USA).

### 2.5 Cytotoxicity Assay in mESCs and 3T3 Cells

Cell viability was assessed using the dimethylthiazolyl diphenyl tetrazolium bromide (MTT) assay. Exponentially growing 3T3 cells and mESCs (500 cells/well each), cultured in the absence of LIF, were seeded into 96‐well microtiter plates. Test compounds (thiram, fluorescein, eosin Y, 2 Mb, 2E4M, DB, and TDA) were added to each well 2 h after cell seeding. The culture medium was replaced on days 3 and 5. The MTT assay was conducted after 10 days of cultivation. Subsequently, 0.5 mg/mL MTT (Sigma‐Aldrich, St. Louis, MO, USA) was added to the medium, and the cells were incubated for an additional 2 h. Formazan crystals were dissolved in 130 μL of DMSO, and absorbance was measured at 570 and 630 nm. Data were expressed as percentages relative to the control. The half‐maximal inhibitory concentration (IC_50_) was determined from dose–response curves, corresponding to 50% inhibition of cell viability in D3 and 3T3 cells (IC_50_D3 and IC_50_3T3, respectively). IC_50_ values were calculated using GraphPad Prism 5 software (GraphPad Software, Boston, MA, USA).

### 2.6 Embryotoxicity Classification of Chemicals

The European Commission's Joint Research Centre (JRC) proposed that the mouse embryonic stem cell test (EST), which evaluates the half‐maximal inhibition of mESC and fibroblast viability, as well as the inhibition of cardiac differentiation of EBs, can be applied to assess the embryotoxic potential of chemicals [36]. EST is based on identifying differences in sensitivity to cytotoxic damage between embryonic tissues (mESCs, D3 cells) and adult tissues (fibroblasts, 3T3 cells). Three endpoints were selected within the EST framework to predict embryotoxicity: inhibition of D3 cell differentiation (ID_50_D3), inhibition of D3 and 3T3 cell viability (IC_50_D3 and IC_50_3T3, respectively). The viability of 3T3 and D3 cells was assessed using the MTT assay, while inhibition of mESC differentiation into contracting cardiomyocytes was evaluated by light microscopy.

The linear discriminant analysis was modified:

- I: 5.916 log (IC_50_3T3) + 3.500 log (IC_50_D3) − 5.307 [(IC_50_3T3‐ID_50_D3)/IC_50_3T3] −15.27
- II: 3.651 log (IC_50_3T3) + 2.394 log (IC_50_D3) − 2.033 [(IC_50_3T3‐ID_50_D3)/IC_50_3T3] −6.85
- III: −0.125 log (IC_50_3T3) − 1.917 log (IC_50_D3) + 1.500 [(IC_50_3T3‐ID_50_D3)/IC_50_3T3] −2.67

Classification criteria:

- Class 1 was not embryotoxic: If I > II and I > III
- Class 2 was weak embryotoxic: If II > I and II > III
- Class 3 was strong embryotoxic: If III > I and III > II

### 2.7 Neuronal Differentiation in mESCs

We differentiated mESCs into neural progenitors and neurons following the protocol described [37]. Neural progenitor cells, which retain stem‐like properties, are capable of differentiating into multiple neural cell types. In the absence of mouse LIF, mESCs underwent spontaneous differentiation. The mESCs (5 × 10^5^ cells) were plated in bacterial‐grade Petri dishes containing 10 mL of serum‐free DMEM medium. For the first 5 days, cells were cultured in the presence of 400 μM L‐proline. From days 6 to 9, L‐proline treatment was continued, and SB431542 was added to promote the differentiation of primitive ectoderm‐like cells into definitive ectoderm and neurectoderm. On day 10, EBs were transferred to gelatin‐coated 6‐well plates containing serum‐free DMEM/F12 medium, and treatment with thiram was initiated. The culture medium was replaced every 2–3 days, and the differentiation process continued until day 17. The experimental procedure for mESC‐derived neuronal differentiation is illustrated in Figure 3A.

### 2.8 Neuronal Differentiation in NT2 Cells

NT2 cells are pluripotent human embryonal carcinoma cells that exhibit stem cell‐like properties [38]. The effects of retinoic acid (RA) or thiram on neuronal differentiation were monitored on day 3 (short‐term model) and day 21 (long‐term model). In the short‐term differentiation model, NT2 cells were exposed to 10 μM RA or thiram for 3 days to induce differentiation. For the long‐term differentiation model, NT2 cells were differentiated into postmitotic neurons as previously described (Megiorni et al., 2005). NT2 cells (5 × 10^5^ cells) were seeded into bacterial‐grade Petri dishes to form floating cell aggregates. After overnight incubation, 10 μM RA or thiram was added to the cultures. The media and dishes were replaced every 3 days. Following 14 days of RA or thiram treatment, the cellular spheres were transferred to tissue culture‐grade 6‐well Petri dishes pre‐coated with 10 μg/mL PDL and 10 μg/mL LAM. The cells were cultured for an additional week in medium supplemented with 10 μg/mL AraC and 1 μg/mL uridine. The medium was replaced every 3 days with fresh AraC‐ and uridine‐supplemented medium. Under these conditions, the aggregates consisted of large, flat cells and numerous neuronal cells with small, bright bodies, from which a complex network of long dendritic and axonal processes developed. The experimental process of NT2‐derived neuronal differentiation is shown in Figure 5A.

### 2.9 Microarray and Bioinformatic Analysis

Total RNA was extracted using the RNeasy Mini Kit (QIAGEN, Hilden, Germany). The quality of the RNA was assessed using a Bioanalyzer 2100 (Agilent Technologies, Santa Clara, CA, USA). Samples were hybridized to Clariom S Assay, human Arrays (Thermo Fisher Scientific, Waltham, MA, USA) and subsequently scanned at the Microarray Core Facility of the National Health Research Institutes (NHRI), Taiwan. The data were analyzed using the Transcriptome Analysis Console Software (Thermo Fisher Scientific, Waltham, MA, USA) with default analysis settings. Expression values were processed using the Signal Space Transformation and Robust Multichip Average (SST‐RMA) method, which included log2 transformation and quantile normalization. The Web‐based GEne SeT AnaLysis Toolkit (https://www.webgestalt.org/) is a widely used tool for functional enrichment analysis. WebGestalt was employed to perform Gene Set Enrichment Analysis (GSEA) for pathway identification.

### 2.10 Real‐Time Quantitative Reverse Transcription Polymerase Chain Reaction Assay

Total RNA was isolated using RNAzol reagent (Life Technologies, Rockville, MD, USA). Complementary DNA (cDNA) was synthesized using the High‐Capacity cDNA Reverse Transcription Kit (Applied Biosystems, Foster City, CA, USA). Polymerase chain reaction (PCR) was conducted using either the TaqMan Universal PCR Master Mix or Power SYBR Green PCR Master Mix (Applied Biosystems, Foster City, CA, USA) on an ABI StepOnePlus Real‐Time PCR System (Perkin‐Elmer Applied Biosystems, Foster City, CA, USA). The assay IDs for all TaqMan assays and the primer sequences for the SYBR Green assays are listed in Supporting Information Tables S1 and S2.

### 2.11 Immunofluorescence (IF) Staining

Cells were seeded in a 6‐well plate. Following treatment and differentiation, the cells were fixed with a methanol/acetone (1:1) solution for 15 min on ice, then washed three times with phosphate‐buffered saline (PBS). Cells were incubated overnight at room temperature with diluted Nestin antibody (Santa Cruz, CA, USA; SC‐23927, 1:200 dilution), Neuronal nuclei (NeuN) antibody (iReal Biotechnology, Hsinchu, Taiwan; IR20‐56, 1:250 dilution), and microtubule associated protein 2 (MAP2) antibody (iReal Biotechnology, Hsinchu, Taiwan; IR12‐33, 1:250 dilution). After washing with PBS, cells were incubated with fluorescein‐conjugated secondary antibodies (Invitrogen, Eugene, OR, USA) at room temperature for 20 min. Fluorescent images were acquired using an Olympus fluorescence microscope (Olympus America Inc., Center Valley, PA, USA).

### 2.12 Western Immunoblotting

NT2 cells were harvested using PRO‐PREP Protein Extraction Solution (iNtRON Biotechnology, Gyeonggi‐do, South Korea) according to the manufacturer's instructions. Protein concentrations were determined using the bicinchoninic acid assay (Pierce Biotechnology, IL, USA). Equal amounts of protein were separated by sodium dodecyl sulfate–polyacrylamide gel electrophoresis and transferred onto polyscreen polyvinylidene difluoride membranes (Bio‐Rad Laboratories, CA, USA). After blocking with 5% skim milk, the membranes were incubated with primary and secondary antibodies, developed using a chemiluminescent HRP substrate, and visualized by exposure to light‐sensitive film (Super RX Fuji medical X‐ray film, Fujifilm Europe GmbH, Dusseldorf, Germany) to detect the target proteins. Cells were stained with anti‐Nanog (1:1000 dilution, Abcam, Waltham, MA, USA) and anti‐glyceraldehyde 3‐phosphate dehydrogenase (GAPDH) (1:100000 dilution, Santa Cruz, CA, USA) antibodies.

### 2.13 Statistical Analysis

Figures were generated using SigmaPlot (version 12.5, Systat Software Inc., San Jose, CA, USA). Statistical analyses were conducted using GraphPad Prism 5 (GraphPad Software, Boston, MA, USA) and SPSS Statistics version 24.0 (IBM SPSS, Chicago, IL, USA). Gene expression data were analyzed using one‐way or two‐way analysis of variance (ANOVA) followed by Tukey's post hoc test in SPSS Statistics, with statistical significance set at *p* < 0.05. Differences in beating rate were assessed using a two‐tailed *t*‐test. The IC_50_ and ID_50_ values of mEST were calculated using GraphPad Prism 5 via nonlinear regression curve fitting with the log (inhibitor) versus normalized response equation.

## 3 Result

### 3.1 In Silico Prediction of Developmental and Reproductive Toxicity (DART)

Following Figure 1, we began assessing the DART toxicity of FCMs using the *in silico* WoE model. The WoE scores of 127 FCMs exceeded 0.5, indicating a high level of toxicity concern (Supporting Information Table S3). The Cramer decision tree, the most widely used Threshold of Toxicological Concern (TTC) approach for non‐cancer endpoints, was applied to further categorize the FCMs. As shown in Table S3, 115 of the 127 FCMs were classified as Class III by the Cramer classification, suggesting a high level of toxicological concern.

### 3.2 Validation of Embryotoxicity of FCMs Using the mEST

Subsequently, we verified four chemicals of high toxicity concern and 3 chemicals of no toxicity concern using the mEST [36]. The mEST evaluated both the differentiation of mESCs and cytotoxicity in mESCs and 3 T3 fibroblast cells to predict embryotoxic potential. The IC_50_ values from cytotoxicity assays in mESCs and 3T3 cells, along with the ID_50_ values from the cardiomyocyte differentiation assay in mESCs, are presented in Table 1. Additionally, we conducted linear discriminant analysis to classify embryotoxicity. Our findings revealed that thiram exhibited strong embryotoxicity, while fluorescein, eosin Y, and 2 Mb displayed weak embryotoxicity among the chemicals of high concern. Among the chemicals of no toxicity concern, 2E4M showed weak embryotoxicity, whereas DB and TDA exhibited no embryotoxicity. These results demonstrate that the WoE model effectively prioritized chemicals with potential embryotoxicity.

**TABLE 1:** The prediction for embryotoxicity of 7 chemicals with mouse embryonic stem cells test (mEST).

	WoE prediction score	3T3 cells	Mouse embryonic stem cells (mESCs)		Prediction embryotoxicity
		IC_50_ (μg/mL)	IC_50_ (μg/mL)	ID_50_ (μg/ml)	
Thiram	0.62	0.04 ± 0.02	0.03 ± 0.01	0.18 ± 0.04	Strong
Fluorescein	0.89	115.35 ± 11.10	91.29 ± 4.96	19.85 ± 0.93	Weak
Eosin Y	0.75	78.21 ± 7.38	90.66 ± 1.05	84.59 ± 9.84	Weak
2,2′‐Methylenebis(4‐methyl‐6‐tert‐butylphenol) (2 Mb)	0.59	16.90 ± 0.08	6.77 ± 0.12	3.52 ± 1.09	Weak
2‐Ethyl‐4‐methylimidazole (2E4M)	−1.00	342.25 ± 25.95	357.60 ± 2.97	175.00 ± 27.86	Weak
Dodecylbenzene (DB)	−1.00	> 8650.00	> 8650.00	> 8650.00	—
1,2,3,6‐Tetrahydrophthalic anhydride (TDA)	−1.00	> 294.00	> 294.00	> 294.00	—

*Note:* The results are presented as mean ± SD for three independent experiments with 8 replicates in each experiment.

### 3.3 Effects of Thiram on Gene Expression of Pluripotent, Endoderm, Mesoderm, Ectoderm, and Cardiomyocyte Markers in mESCs

Based on our results, thiram posed a significant concern for embryotoxicity. Therefore, we selected thiram for further investigation into its mechanisms of embryonic developmental toxicity. We first examined how thiram interfered with the development of the three germ layers in mESCs. Previous studies reported that the expression of *pluripotency genes like octamer‐binding protein 4* (*Oct4*), *Nanog*, and *sry‐box containing gene 2* (*Sox2*) decreases, while the expression of germ layer‐specific genes such as *Brachyury T*, *GATA‐binding factor 6* (*Gata6*), *fibroblast growth factor 5* (*Fgf5*), *proenkephalin1* (*Penk1*), and *sry‐box containing gene 1* (*Sox1*) increases during EB differentiation [39, 40]. In our study, the gene expression changes observed during EB differentiation were consistent with those reported in the literature. When mESCs were treated with 0.15 μg/mL thiram (approximately the ID_50_ dose) for 3 days, the mRNA levels of the pluripotency markers *Sox2* and *Oct4* remained unchanged (Figure 2A). However, the mRNA level of *Nanog* was decreased (Figure 2A). Thiram reduced the mRNA levels of early ectodermal markers (*Fgf5* and *Penk1*) and the early mesodermal marker (*Brachyury T*) but did not reduce the expression of the early endodermal marker *Gata6* (Figure 2B). In contrast, thiram increased the expression of neurectodermal markers (*Sox1* and *homeobox A1, Hoxa1*) during the early stages of embryogenesis (Figure 2C). Additionally, these effects were time‐dependent (Supporting Information Figure S1). These findings indicated that thiram interfered with the differentiation of mESCs into the three germ layers.

**FIGURE 2:**
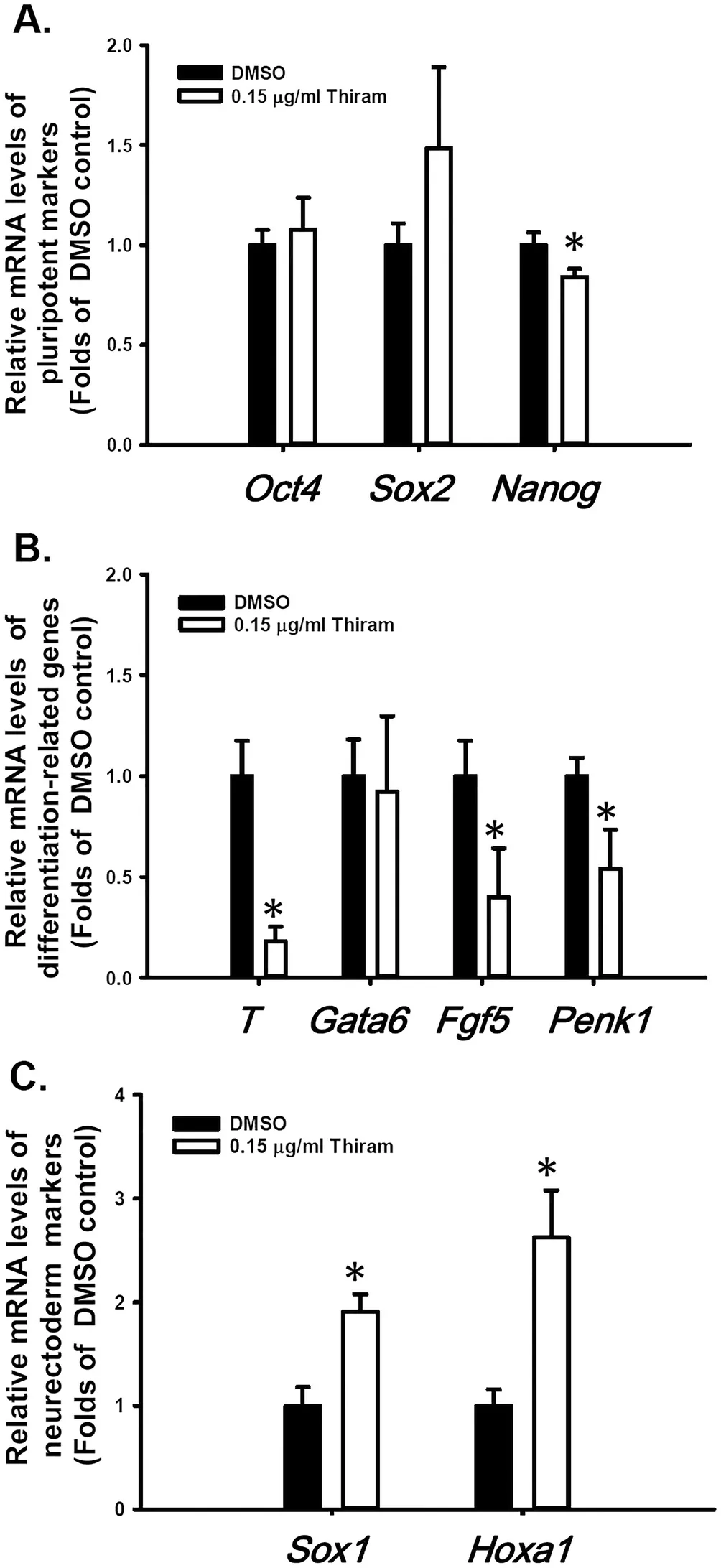
Effect of thiram on the expression of differentiation‐related genes in mouse ESCs. mESCs were treated with DMSO or 0.15 μg/mL thiram for 3 days during cardiomyocyte differentiation. Differentiation markers included genes associated with (A) pluripotent stem cells, (B) mesoderm, ectoderm, and endoderm, and (C) neuroectoderm. Data are presented as the mean ± standard deviation (SD) from three independent experiments, each with three replicates. *p < 0.05, compared with DMSO‐treated cells.

### 3.4 Effect of Thiram on Neuronal Differentiation in mESCs

Based on the above results, thiram increased the expression of neurectodermal markers (*Sox1* and *Hoxa1*) in mESCs. Therefore, we investigated whether thiram affected the differentiation of neural progenitors in mESCs. The mESCs are pluripotent and can differentiate in vitro into various cell types, including neural cells, making them a valuable model for studying the mechanisms of mammalian embryonic development.

The experimental process of neuronal differentiation from mESCs, including progression to neural progenitors and neurons, was illustrated in Figure 3A. Retinoic acid (RA), a known inducer of developmental neurotoxicity (DNT), was used as a positive control [41]. RA significantly increased the mRNA expression levels of the neural progenitor marker *Nestin* and the neuronal marker *NeuN* on day 17 of mESC‐derived neuronal differentiation (Figure 3B). Similarly, treatment with 20 ng/mL thiram, which was the lowest‐observed effect‐concentration (LOEC) of cytotoxicity, significantly elevated *Nestin* and *NeuN* mRNA levels in a dose‐dependent manner (Figure 3C). Neurite outgrowth, a critical process in early neuronal development, serves as a key in vitro indicator of DNT [42]. The addition of thiram to the culture medium significantly enhanced neurite outgrowth on day 17 (Figure 3D). These findings indicated that thiram promoted neuronal differentiation in mESCs, suggesting its potential impact during early embryonic development.

**FIGURE 3:**
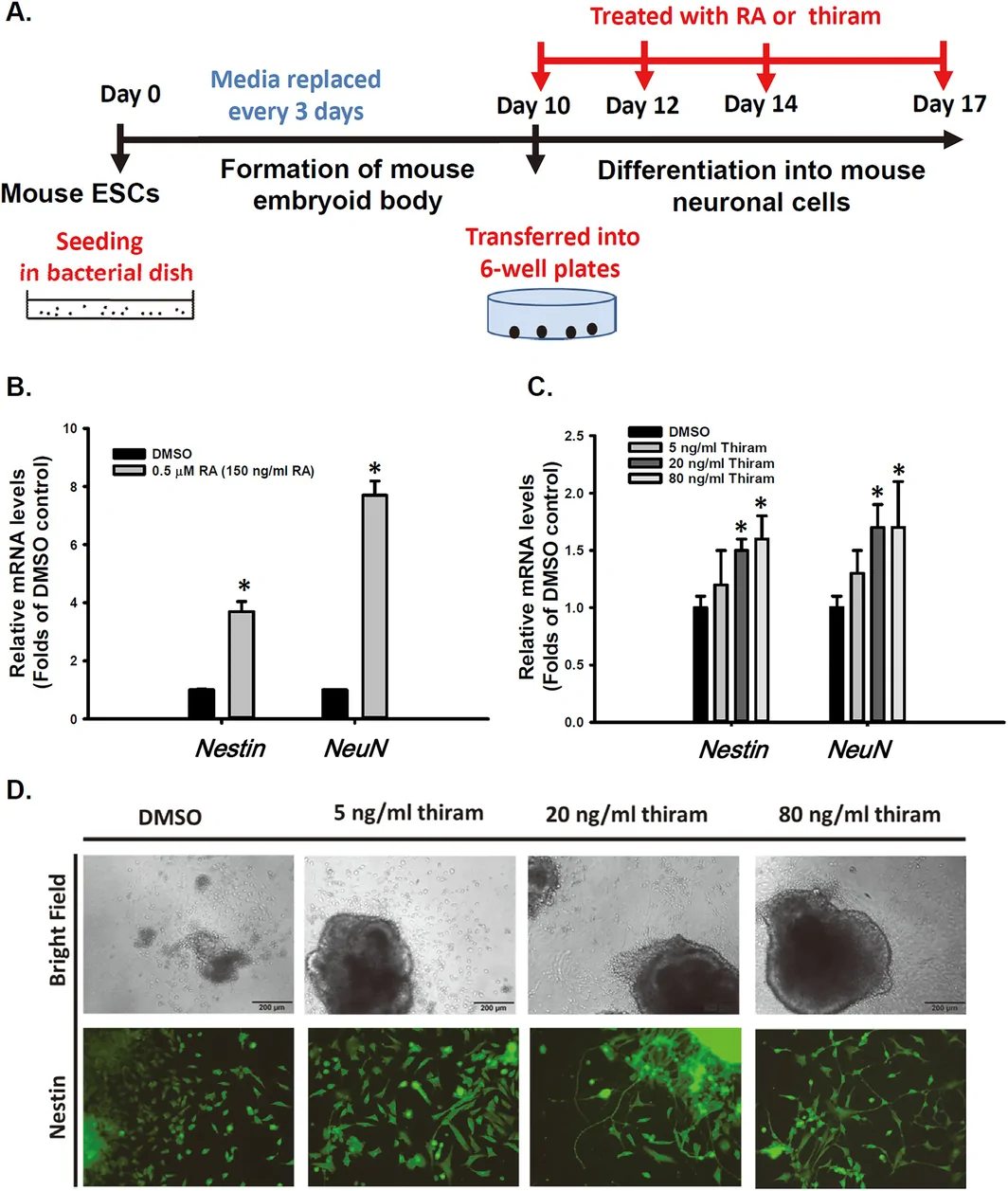
Effect of thiram on neuronal differentiation in mouse ESCs. (A) Schematic of the experimental workflow for mESC‐derived neuronal differentiation. mESCs were treated with DMSO, 5, 20, 80 ng/mL thiram, or 0.5 μM retinoic acid (RA) during neuronal differentiation. Expression of neuronal differentiation markers was measured using real‐time PCR. (B) Effects of RA treatment. (C) Effects of thiram treatment. Data are presented as the mean ± SD from three independent experiments, each with three replicates. *p < 0.05, compared with DMSO‐treated cells. (D) Morphology and Nestin protein expression of mESCs on day 17, visualized by immunofluorescence (IF) staining. Scale bar, 200 μm.

### 3.5 Effect of Thiram on Neuronal Differentiation in Human NT2 Cells

To address species‐specific differences between mice and humans, we investigated whether thiram disrupted neuronal differentiation in human NT2 cells. NT2 cells are increasingly recognized as a relevant in vitro model for assessing developmental toxicity (DT) and DNT [43, 44]. Menzner et al. (2015) demonstrated that short‐term (3 days) differentiation assays in NT2 cells could reliably assess chemical susceptibility for in vitro developmental neurotoxicity testing. We first evaluated the cytotoxicity of thiram using a 3 days short‐term differentiation model (Figure 4A). The IC_50_ of thiram in NT2 cells was 52.55 μg/mL (Figure 4B). 10 μM RA reduced the mRNA expression of pluripotency markers *OCT4*, *NANOG*, and *SOX2*, whereas 20 and 30 ng/mL thiram decreased only *NANOG* mRNA levels after 3 days of treatment (Figure 4C,D). Additionally, treatment with both RA and thiram resulted in decreased NANOG protein levels (Figure 4E). Furthermore, RA increased mRNA expression of *NESTIN*, a neural progenitor marker, but decreased *NEUN*, a neuronal marker. In contrast, thiram increased only *NESTIN* mRNA levels, but did not affect *NEUN* mRNA levels (Figure 4C,D). Consistently, IF staining demonstrated that both RA and thiram elevated NESTIN protein levels in NT2 cells (Figure 4F). These findings indicated that both RA and thiram interfered with early neuronal differentiation within 3 days in NT2 cells.

**FIGURE 4:**
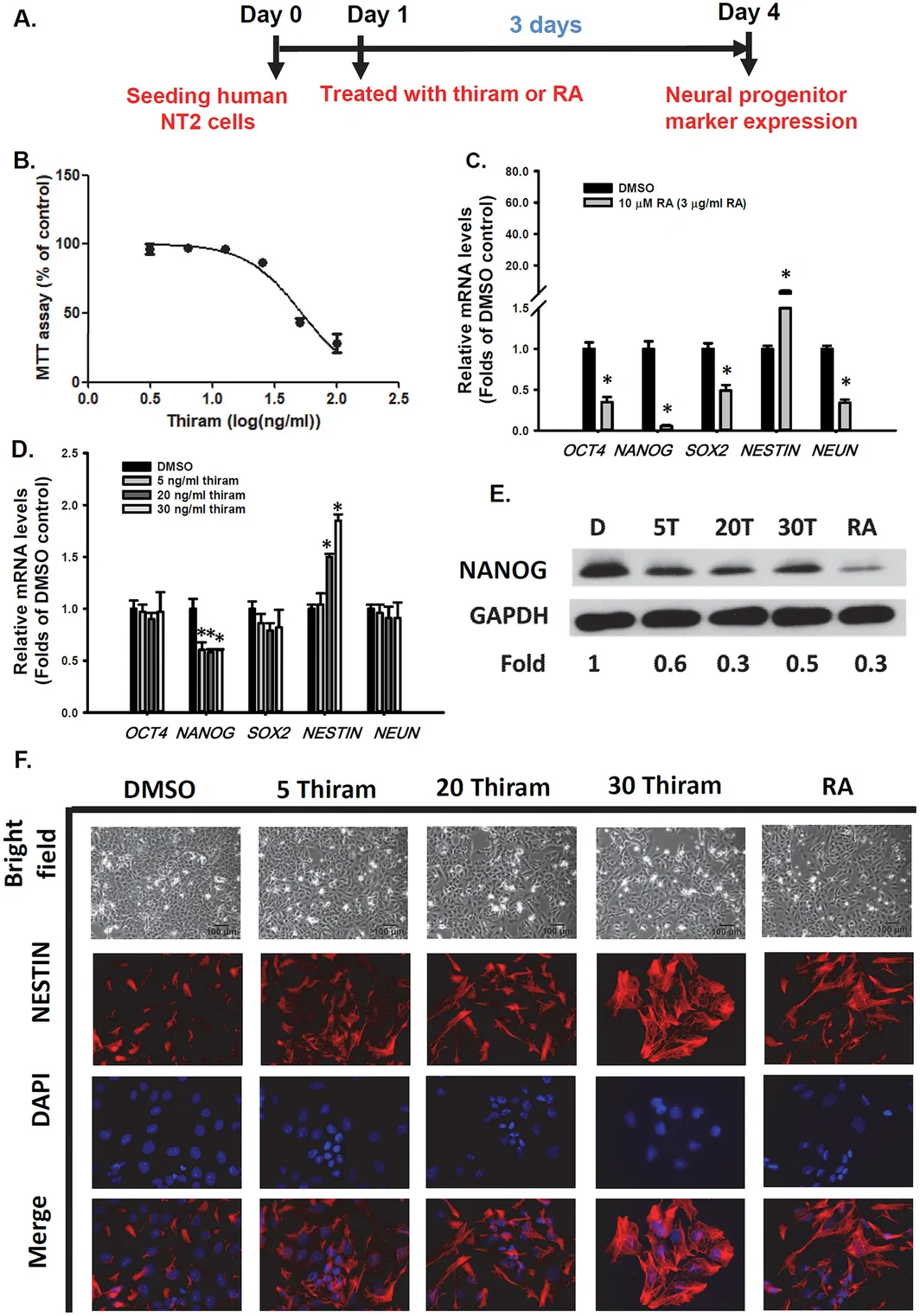
Effect of thiram on a short‐term neuronal differentiation model in human NT2 cells. (A) Schematic of the experimental workflow for NT2‐derived short‐term neuronal differentiation. NT2 cells were treated with DMSO, 5, 20, 30 ng/mL thiram, or 10 μM RA for 3 days in a short‐term neuronal differentiation model. (B) Cell viability was assessed using the MTT assay. Gene expression of neuronal differentiation markers was measured using real‐time PCR. (C) Effects of RA treatment. (D) Effects of thiram treatment. Data are presented as the mean ± SD from three independent experiments, each with three replicates. *p < 0.05, compared with DMSO‐treated cells. (E) Nanog protein expression in NT2 cells on day 17, evaluated by western blotting. (F) Morphology and Nestin protein expression in NT2 cells on day 17, visualized by IF staining. Scale bar, 100 μm.

NT2 cells are capable of generating postmitotic neurons and serve as a valuable in vitro model for studying human neurogenesis [43]. Neurons constitute the fundamental structural and functional units of the nervous system, responsible for receiving, processing, and transmitting nerve signals. To investigate long‐term differentiation, we conducted an extended 21 days differentiation study on NT2 cells, as illustrated in Figure 5A. RA (0.1 and 0.5 μM) and thiram (20 and 30 ng/mL) treatment significantly reduced the mRNA levels of pluripotent stem cell markers *OCT4* and *NANOG* by day 21 during NT2 cell‐derived neuronal differentiation (Figure 5B,C). Furthermore, both RA and thiram downregulated *NESTIN* mRNA levels while upregulating the expression of neuronal markers *NEUN* and *MAP2* (Figure 5B,C). IF staining also demonstrated that RA and thiram increased NeuN and MAP2 protein expression levels in NT2 cells (Figure 5D). Additionally, both compounds promoted neurite outgrowth (Figure 5D). These results aligned with observations from the short‐term differentiation model, indicating that thiram similarly interfered with neuronal differentiation in the long‐term model.

**FIGURE 5:**
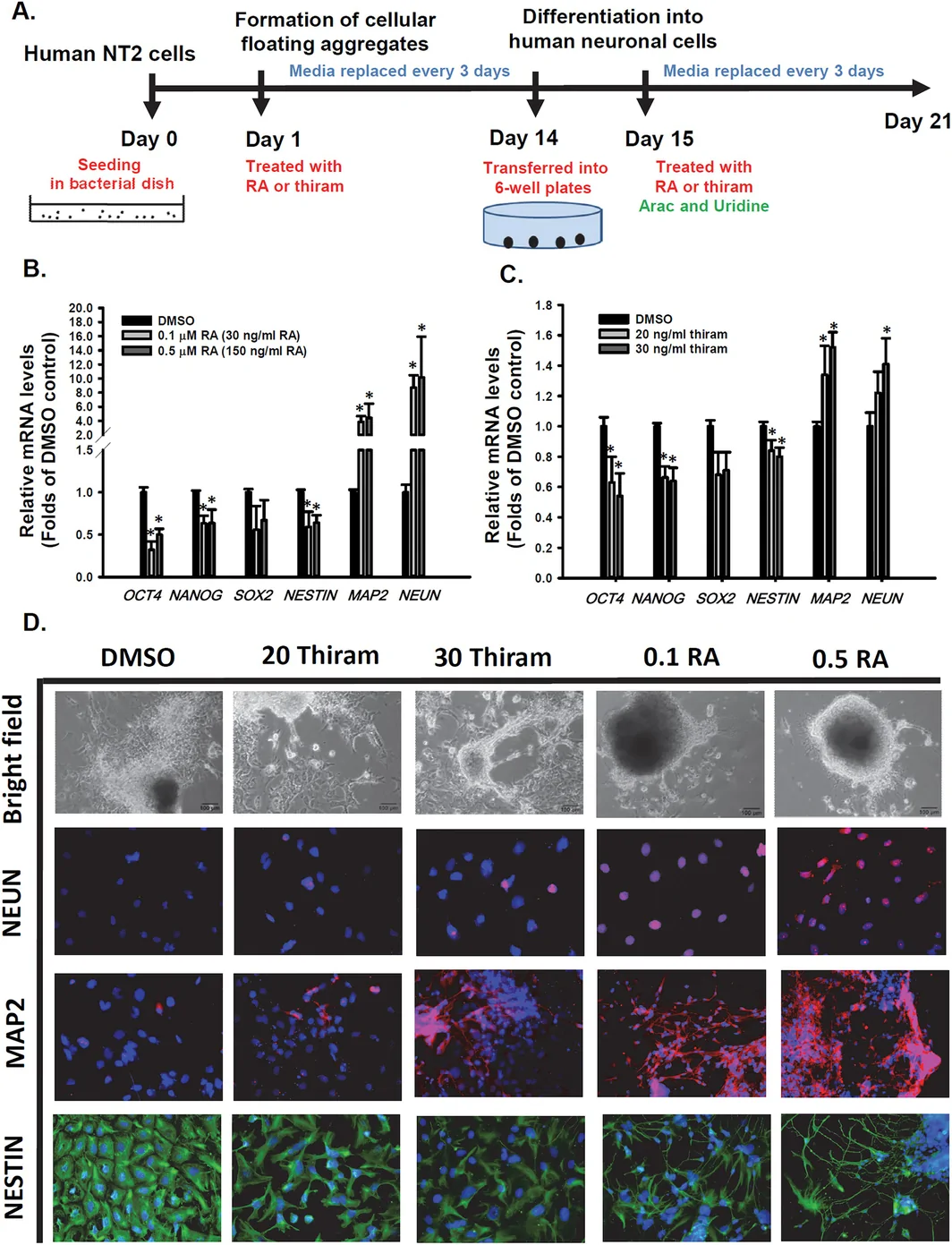
Effect of thiram on a long‐term neuronal differentiation model in human NT2 cells. (A) Schematic of the experimental workflow for NT2 cell‐derived neuronal differentiation. NT2 cells were treated with DMSO, 20 or 30 ng/mL thiram, or 0.1 or 0.5 μM RA in a long‐term differentiation model. Gene expression of neuronal differentiation markers was measured using real‐time PCR. (B) Effects of RA treatment. (C) Effects of thiram treatment. Data are presented as the mean ± SD from three independent experiments, each with three replicates. *p < 0.05, compared with DMSO‐treated cells. (D) Morphology and expression of Nestin, NeuN, and MAP proteins in NT2 cells on day 21, visualized by IF staining. Scale bar, 100 μm.

### 3.6 Transcriptomic Analysis for Thiram‐Treated NT2 Cells

To identify early biomarkers affected by thiram during early embryonic development, transcriptomic analysis was conducted on thiram‐treated NT2 cells. We examined the effects of 10 μM RA, as well as 20 and 30 ng/mL thiram on the genome‐wide gene expression profile of NT2 cells following a 3‐day treatment (Figure 6A). A total of 297 genes were differentially expressed between cells treated with 30 ng/mL thiram and those treated with 0.1% DMSO. The threshold for differential gene expression was set at a twofold change. Among the 297 differentially expressed genes, 218 overlapped with RA‐induced genes (Figure 6B,C), suggesting that thiram may elicit toxicological mechanisms similar to those of RA. GSEA was performed with a focus on the pathway functional database. In the WikiPathways functional database, the top upregulated pathways in response to thiram treatment included neural crest differentiation, vitamin A and carotenoid metabolism, nuclear receptors in lipid metabolism and toxicity, mesodermal commitment, and TGF‐β receptor signaling (Figure 6D). Thiram altered the expression of 23 genes within the neural crest differentiation pathway. Among these, the expression of seven genes was further validated using RT‐PCR. As shown in Figure 6E, treatment with 10 μM RA for 3 days significantly increased the relative mRNA levels of *HOXA1, Homeobox b1 (HOXB1), Heart And Neural Crest Derivatives Expressed 1 (HAND1), Insulin gene enhancer protein ISL‐1 (ISL1), Bone morphogenetic protein 4(BMP4), Distal‐Less Homeobox 5 (DLX5)* and *transcription factor AP‐2 alpha (TFAP2A)*. Thiram treatment produced a similar dose‐dependent increase in the relative mRNA levels of *HOXA1*, *HOXB1*, *HAND1*, *DLX5*, and *TFAP2A*, except for *ISL1* or *BMP4* expression in NT2 cells (Figure 6F). These results indicated that thiram disrupted the neural crest differentiation pathway in NT2 cells, mirroring the effects observed with RA treatment.

**FIGURE 6:**
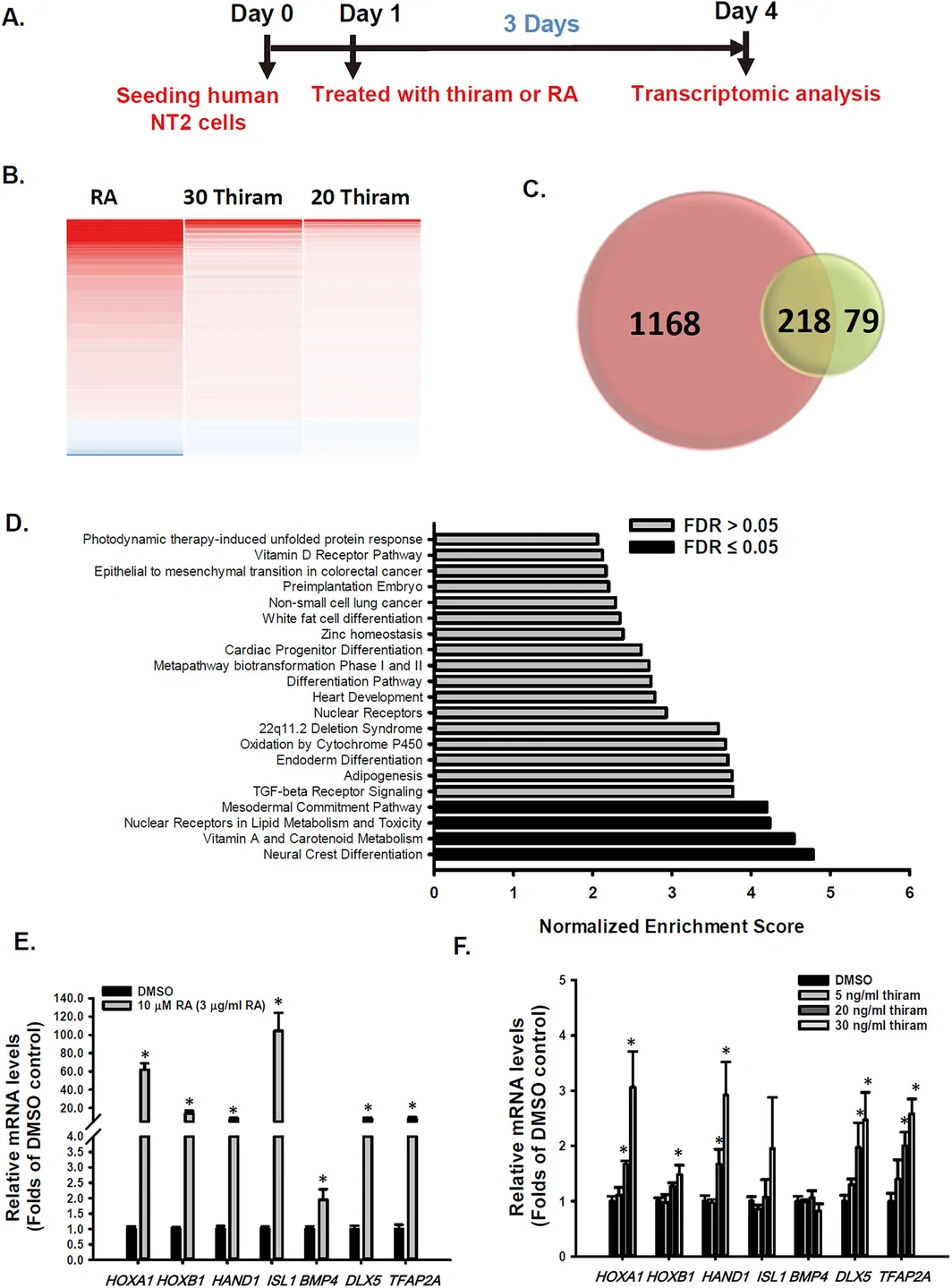
Effect of thiram on the transcriptomic profile in human NT2 cells. (A) Schematic of the experimental workflow for NT2‐derived short‐term neuronal differentiation. (B) Heat map of differential expression between RA, 20 and 30 ng/mL thiram‐treated NT2 cells. (C) Overlap in differential gene expression after RA and thiram exposure. (D) GSEA of transcripts from NT2 cells treated with 30 ng/mL thiram for 3 days. Normalized enrichment scores for the top regulated pathways are shown and categorized according to WikiPathways. (E) Effects of RA treatment. (F) Effects of thiram treatment. Gene expression was measured using real‐time PCR. Data are presented as the mean ± SD from three independent experiments, each with three replicates. *p < 0.05, compared with DMSO‐treated cells.

## 4 Discussion

Based on the 3R principles, in vitro and in silico methods have been applied to assess the safety of chemicals rapidly under limited toxicological data. Although a few studies have shown that thiram could cause developmental toxicity in mice, rats, poultry, and zebrafish embryos, mechanistic evidence generated in human‐relevant models remains lacking. In the present study, a combination of in silico analysis, mouse and human in vitro models, and transcriptomic profiling was employed to validate the embryotoxicity in animal studies and elucidate the embryotoxic mechanisms of thiram. We found that thiram interfered with the differentiation of mESCs into the three germ layers. Prolonged treatment with thiram (17–21 days) disrupted neuronal differentiation in both mESCs and human NT2 cells, suggesting that thiram impairs early embryonic development. Transcriptomic analysis further revealed that 3‐day thiram treatment disrupted the neural crest differentiation pathway in NT2 cells, altering the expression of key genes such as *HOXA1*, *HOXB1*, *HAND1*, *DLX5*, and *TFAP2A*. Based on these findings, we propose that disruption of neural crest differentiation is a potential mechanism underlying thiram‐induced embryotoxicity. Furthermore, our study establishes an integrated framework that integrates *in silico* prediction with in vitro validation to enable safety assessment of data‐poor chemicals in the future.

Thiram has been associated with multiple toxic effects, including thyroid disruption [21], in addition to developmental, hepatic, and neuronal toxicity in animals [15, 22, 23]. Consistent with our findings, previous studies have shown that thiram can induce developmental abnormality in both aquatic and mammalian models. Thiram has also been shown to induce developmental defects, such as growth retardation (1–10 μM), heart malformation (1–10 μM), delayed hatching (0.1–1 μM), abnormal craniofacial development (0.1–1 μM), and spinal curvature (0.1–1 μM) in zebrafish embryos [21, 45]. In mammal models, prenatal exposure led to fetal malformations. Mice exposed to 1200 mg/kg/day from gestational days 6–17 exhibited cleft palate, wavy ribs, and long‐bone deformities [46], whereas rats showed delayed skull ossification [15]. In hamsters, maternal doses of 125 mg/kg/day were teratogenic, resulting in incomplete formation of the skull and spine, fused ribs, and malformation of limbs and the heart [46]. Thiram could also inhibit egg production in poultry, with effects observed at concentrations of approximately 6.8 mg/kg/day [47] in chickens and 8.8 mg/kg/day in quails [48]; it could also affect partridges. In addition, thiram has been reported to interfere with TGF‐β/SMAD signaling and neurite outgrowth in iPSC‐derived neurons [49]. However, in our NT2 cell studies, treatment with similar concentrations (approximately 120 ng/mL) resulted in cell death (data not shown). Conversely, lower doses (20–30 ng/mL) of thiram disrupted neural crest differentiation, mesodermal commitment pathway, and TGF‐β receptor signaling in NT2 cells. Such in vivo findings, together with our in vitro data in mESCs and human NT2 cells, suggest that thiram interferes with early embryonic patterning and neural lineage differentiation.

From an environmental perspective, the annual global DTC fungicide usage is estimated to be 25 000–35 000 tons [17]. Thiram, one of the most widely used DTC fungicides, has a half‐life of approximately 15 days in soil [50]. Residual thiram in soil can leach into groundwater, polluting water resources, with concentrations in water reported to range from 0.27 to 2.52 μg/mL (≈1.1–10.5 μM) following application in oil palm nurseries [51]. The estimated environmental concentration (EEC) of thiram in surface water in the USA was approximately 4.3 ng/mL (0.018 μM), whereas groundwater EEC was estimated to be 0.84 ng/mL (0.004 μM) [20]. Therefore, humans are likely to experience continuous low‐level exposure through dietary intake or environmental contact. However, DTC fungicides are transformed rapidly into ethylenethiourea (ETU) in the environment, and the DTC fungicide and ETU residues in foodstuffs are measured indirectly as carbon disulfide. The degradation product of DTC fungicide, ETU, has been detected in human biological samples, including the urine of pregnant women and children [52].

Recently, researchers and regulatory agencies have been attempting to apply the new methodology adopted in the present study in regulatory decision‐making. For example, integration of the in vitro–in vivo extrapolation (IVIVE) and physiological‐based pharmacokinetic (PBPK) models would convert in vitro concentrations into equivalent administrated dose (EAD) in humans [53, 54, 55, 56]. In addition, the Integrated Chemical Environment (ICE), developed by the National Toxicology Program, is an open‐access web tool that provides IVIVE and PBPK model workflows [57, 58]. In the present study, an IVIVE‐human fetal PBPK model in ICE version 4.2, released on July 2025 (https://ice.ntp.niehs.nih.gov/), was used to estimate tissue‐specific concentrations after administration of certain doses. Subsequently, the human EAD correlated to in vitro LOEC was calculated. Considering a pregnant woman to whom 1 mg/kg/day thiram is administered for 7 days, according to the PBPK model the maternal and fetal plasma thiram concentrations would be 24.2 and 25.7 μM, respectively. In the present study, the LOEC in NT2 cells was 20 ng/mL thiram. If the steady state concentration was linear with the oral dose, the IVIVE model estimated that 20 ng/mL in NT2 cells was correlated to a human EAD of 0.003 mg/kg/day. The estimated EAD was comparable to the acceptable daily intake (ADI) for thiram (0.004 mg/kg/day) [59]. In summary, the in vitro LOEC determined in the present study is comparable to the ADI, which is the safety threshold in humans. To date, however, most safety thresholds for chemical regulation have been calculated based on the no‐observed‐adverse‐effect level in animal studies, without considering mechanistic information. The present study offers mechanistic evidence in a human‐relevant model at the human EAD close to the safety threshold, ADI. Such information could enhance the confidence of stakeholders with regard to establishment of safety thresholds based on alternative approaches in future. In addition, mechanism‐based early biomarkers for developmental toxicity would facilitate determination of the values of acceptable risk in humans by regulatory agencies.

Neural crest cells (NCCs) are a population of multipotent, migratory cells in vertebrate embryogenesis that contribute to the formation and differentiation of various cell types, including neurons, support cells of the peripheral nervous system, melanocytes, craniofacial cartilage and bone, smooth muscle cells of the cardiovascular system, and endocrine cells [60, 61, 62]. The progression of neural crest differentiation involves a complex network of transcription factors and downstream target genes [63]. Hox genes, such as HOXA and HOXB, play critical roles at multiple stages of cranial neural crest cell (cNCC) development, including specification, migration, and differentiation [64, 65]. DLX5 is expressed during the differentiation of NCC derivatives, including cranial nerve ganglia, cartilage, and bone in mice [66]. Jaw formation in mice depends on the coordinated expression of Dlx5 in cNCCs and ectodermal regions [67]. HAND1 is essential for NCC differentiation, playing a major role in cardiac and craniofacial development by regulating both structure and function [68]. TFAP2A is a key transcription factor involved in NCC development, promoting their differentiation into melanocytes in zebrafish [64, 69, 70]. In the present study, we demonstrated that thiram induced the mRNA expression of *HOXA1*, *HOXB1*, *HAND1*, *DLX5*, and *TFAP2A* in NT2 cells. Based on these findings, we suggest that thiram disrupts the neural crest differentiation pathway during early embryonic development.

Several studies have investigated the effects of thiram exposure on zebrafish embryonic development. Thiram was found to alter the expression pattern of the sox9a gene, a key regulator of neural crest specification, in zebrafish embryos [45]. Additionally, thiram induced craniofacial abnormalities due to impaired chondrocyte development and caused bradycardia in zebrafish embryos [18, 21, 45, 71]. Abnormal NCC development has been associated with cardiovascular abnormalities and craniofacial malformations in zebrafish embryo [72], mouse embryo [73, 74, 75], and human [76]. Based on these findings, we propose that thiram may interfere with neural crest differentiation pathways. However, further mechanistic studies are required to confirm this hypothesis.

Many pesticides target the nervous systems of pests and may also be neurotoxic to humans, potentially impacting embryonic development. However, current safety testing requirements do not include DNT assessments [77]. In vitro alternative methods can offer preliminary evaluations and assist in prioritizing chemicals that may induce DNT. These approaches are essential for elucidating mechanisms of toxicity—critical for risk assessment—and are often difficult to obtain through in vivo studies alone [78]. The DT/DNT test in NT2 cells integrates multiple methodologies across various stages of NT2 cell development. These include assessments of nonspecific cytotoxicity, analyses of neuronal marker expression, identification of chemicals disrupting intracellular signaling pathways, and morphological evaluations of neuronal network formation [79, 80, 81]. In our study, we evaluated thiram‐induced cytotoxicity, neuronal marker expression, and toxicity mechanisms during neuronal differentiation using the DT/DNT test in NT2 cells. This model may be applied in future research to assess the potential effects of pesticides on early embryonic development.

## Author Contributions

Conceptualization: Pinpin Lin, Chia‐Chi Ho. Methodology development: Pinpin Lin, Chia‐Chi Ho, B. Linju Yen. Performing experiments: Chia‐Chi Ho, Chen‐Yi Weng, Ming‐Hsien Tsai. In silico prediction analysis: Chun‐Wei Tung, Ju‐Hsin Hs. Writing – original draft: Pinpin Lin, Chia‐Chi Ho, Salim Arrokhman. Funding Acquisition: Pinpin Lin. All authors have read and approved the final manuscript.

## Funding

This work was supported by the National Health Research Institutes, Taiwan (Grant No. NHRI‐114 EMPP02) and the National Science and Technology Council, Taiwan (Grant No. 111‐2320‐B‐400‐011‐MY3).

## Conflicts of Interest

The authors declare no conflicts of interest.

## Supporting information


**Table S1:** The RT‐PCR was performed with the following TaqMan assay.
**Table S2:** The RT‐PCR was performed with the following SYBR green primers.
**Table S3:** The WoE scores of 127 FCMs.
**Figure S1:** Effect of thiram on expression of differentiation‐related genes in mESCs. The mESCs were treated with DMSO or 0.15 μg/mL thiram for 3–10 days in mESCs‐derived cardiomyocyte differentiation. Differentiation markers included genes for (A) Oct4, (B) Gata6, (C) Fgf5 (D) Brachyury T, and (E) Tnnt2. The results were presented as mean ± standard deviation (SD) of one independent experiment with 3 replicates in each experiment. *p < 0.05, compared with DMSO‐treated cells.

## Data Availability

The data that support the findings of this study are available from the corresponding author upon reasonable request.

## References

[1] J. Muncke , T. Backhaus , B. Geueke , et al., “Scientific Challenges in the Risk Assessment of Food Contact Materials,” Environmental Health Perspectives 125, no. 9 (2017): 095001. 10.1289/EHP644 PMC5915200 28893723

[2] E. N. Pieke , K. Granby , B. Teste , J. Smedsgaard , and G. Riviere , “Prioritization Before Risk Assessment: The Viability of Uncertain Data on Food Contact Materials,” Regulatory Toxicology and Pharmacology 97 (2018): 134–143. 10.1016/j.yrtph.2018.06.012 29932981

[3] A. E. Turley , K. K. Isaacs , B. A. Wetmore , A. L. Karmaus , M. R. Embry , and M. Krishan , “Incorporating New Approach Methodologies in Toxicity Testing and Exposure Assessment for Tiered Risk Assessment Using the RISK21 Approach: Case Studies on Food Contact Chemicals,” Food and Chemical Toxicology 134 (2019): 110819. 10.1016/j.fct.2019.110819 PMC7429715 31545997

[4] M. Van Bossuyt , E. Van Hoeck , T. Vanhaecke , V. Rogiers , and B. Mertens , “Prioritizing Substances of Genotoxic Concern for In‐Depth Safety Evaluation Using Non‐Animal Approaches: The Example of Food Contact Materials,” Alternatives to Animal Experimentation 36, no. 2 (2019): 215–230. 10.14573/altex.1810011 30488084

[5] M. B. Bracken , “Why Animal Studies Are Often Poor Predictors of Human Reactions to Exposure,” Journal of the Royal Society of Medicine 102, no. 3 (2009): 120–122. 10.1258/jrsm.2008.08k033 PMC2746847 19297654

[6] U.S.NRC , Applications of Toxicogenomic Technologies to Predictive Toxicology and Risk Assessment (US National Academies Press, 2007). 20669432

[7] D. Krewski , M. E. Andersen , M. G. Tyshenko , et al., “Toxicity Testing in the 21st Century: Progress in the Past Decade and Future Perspectives,” Archives of Toxicology 94, no. 1 (2020): 1–58. 10.1007/s00204-019-02613-4 31848664

[8] E. Berggren , A. White , G. Ouedraogo , et al., “Ab Initio Chemical Safety Assessment: A Workflow Based on Exposure Considerations and Non‐Animal Methods,” Computational Toxicology 4 (2017): 31–44. 10.1016/j.comtox.2017.10.001 PMC5695905 29214231

[9] OECD , “Guidance Document on the Reporting of Defined Approaches to be Used Within Integrated Approaches to Testing and Assessment, No. 255, ENV/JM/MONO(2016)28,” (2016).

[10] OECD , “Series on Testing and Assessment No. 329: Overview of Concepts and Available Guidance Related to Integrated Approaches to Testing and Assessment (IATA),” in Environment, Health and Safety, Environment Directorate (OECD, 2020).

[11] SCCS , “SCCS Notes of Guidance for the Testing of Cosmetic Ingredients and Their Safety Evaluation 12th Revision, SCCS/1647/22,” (2023). 10.1016/j.yrtph.2021.105052 34653552

[12] C. W. Tung , H. J. Cheng , C. C. Wang , S. S. Wang , and P. Lin , “Leveraging Complementary Computational Models for Prioritizing Chemicals of Developmental and Reproductive Toxicity Concern: An Example of Food Contact Materials,” Archives of Toxicology 94, no. 2 (2020): 485–494. 10.1007/s00204-019-02641-0 31897520

[13] IARC , The Rubber Industry. IARC Monographs on the Evaluation of Carcinogenic Risks to Humans, vol. 28 (International Agency for Research on Cancer, 1982), 1–486.

[14] U.S.FDA , “Inventory of Food Contact Substances Listed in Title 21 of the U.S. Code of Federal Regulations,” (2021), https://www.fda.gov/food/packaging‐food‐contact‐substances‐fcs/inventory‐food‐contact‐substances‐listed‐21‐cfr.

[15] EPA US , Thiram (Agency USEP, 2004).

[16] E. Yuzbasioglu and E. Dalyan , “Salicylic Acid Alleviates Thiram Toxicity by Modulating Antioxidant Enzyme Capacity and Pesticide Detoxification Systems in the Tomato ( *Solanum lycopersicum* Mill.),” Plant Physiology and Biochemistry 135 (2019): 322–330. 10.1016/j.plaphy.2018.12.023 30599309

[17] C. Campanale , M. Triozzi , A. Ragonese , D. Losacco , and C. Massarelli , “Dithiocarbamates: Properties, Methodological Approaches and Challenges to Their Control,” Toxics 11, no. 10 (2023): 851. 10.3390/toxics11100851 PMC10610574 37888701

[18] B. M. K. Vasamsetti , K. Chon , J. Kim , J. A. Oh , C. Y. Yoon , and H. H. Park , “Developmental Toxic Effects of Thiram on Developing Zebrafish ( *Danio rerio* ) Embryos,” Toxics 10, no. 7 (2022): 369. 10.3390/toxics10070369 PMC9317679 35878274

[19] “Estimated Annual Agricultural Pesticide Use, Pesticide Use Maps—Thiram,” (2024), accessed 2018, https://water.usgs.gov/nawqa/pnsp/usage/maps/show_map.php?year=2017&map=THIRAM&hilo=L&disp=Thiram.

[20] EPA U , Reregistration Eligibility Decision for Thiram, Vol EPA 738‐R‐04‐012 (United States Environmental Protection Agency, Office of Protection, Pesticides and Toxic Substances, 2004).

[21] X. Chen , M. Fang , M. Chernick , et al., “The Case for Thyroid Disruption in Early Life Stage Exposures to Thiram in Zebrafish ( *Danio rerio* ),” General and Comparative Endocrinology 271 (2019): 73–81. 10.1016/j.ygcen.2018.11.003 30408483

[22] Canada H , Thiram (Health Canada Pest Management Regulatory Agency, 2016).

[23] K. Liu , Y. Li , M. Iqbal , Z. Tang , and H. Zhang , “Thiram Exposure in Environment: A Critical Review on Cytotoxicity,” Chemosphere 295 (2022): 133928. 10.1016/j.chemosphere.2022.133928 35149006

[24] E. J. Perkins , R. Ashauer , L. Burgoon , et al., “Building and Applying Quantitative Adverse Outcome Pathway Models for Chemical Hazard and Risk Assessment,” Environmental Toxicology and Chemistry 38, no. 9 (2019): 1850–1865. 10.1002/etc.4505 PMC6771761 31127958

[25] National Academies of Sciences E, and Medicine , ed., Using 21st Century Science to Improve Risk‐Related Evaluations (National Academies Press, 2017). 28267305

[26] A. L. Luz and E. J. Tokar , “Pluripotent Stem Cells in Developmental Toxicity Testing: A Review of Methodological Advances,” Toxicological Sciences 165, no. 1 (2018): 31–39. 10.1093/toxsci/kfy174 PMC6111785 30169765

[27] Y. Li , J. Zhang , Y. Zhang , et al., “Integrated Metabolomic and Transcriptomic Analysis Reveals Perturbed Glycerophospholipid Metabolism in Mouse Neural Stem Cells Exposed to Cadmium,” Ecotoxicology and Environmental Safety 264 (2023): 115411. 10.1016/j.ecoenv.2023.115411 37660531

[28] U.S.FDA , “Indirect Additives Used in Food Contact Substances,” (2019), accessed 9 May 2021, https://wwwcfsanappsexternalfdagov/scripts/fdcc/?set=IndirectAdditives.

[29] Commission TE , “Plastic Materials and Articles Intended to Come Into Contact With Food,” in ANNEX I: Substances Official Journal of the European Union (EuropeanUnion, 2011).

[30] FDHA , Annex 10 of the Ordinance of the FDHA on Materials and Articles Intended to Come Into Contact With Foodstuffs (Swiss Federal Department of Home Affairs, 2020).

[31] T. W. Schultz , R. Diderich , C. D. Kuseva , and O. G. Mekenyan , “The OECD QSAR Toolbox Starts Its Second Decade,” Methods in Molecular Biology 1800 (2018): 55–77. 10.1007/978-1-4939-7899-1_2 29934887

[32] C. W. Tung and S. S. Wang . “ChemDIS 2: an Update of Chemical‐disease Inference System,” Database (Oxford) (2018): 2018. 10.1093/database/bay077 PMC6057493 30053236

[33] M. Ashburner , C. A. Ball , J. A. Blake , et al., “Gene Ontology: Tool for the Unification of Biology. The Gene Ontology Consortium,” Nature Genetics 25, no. 1 (2000): 25–29. 10.1038/75556 PMC3037419 10802651

[34] W. A. Kibbe , C. Arze , V. Felix , et al., “Disease Ontology 2015 Update: An Expanded and Updated Database of Human Diseases for Linking Biomedical Knowledge Through Disease Data,” Nucleic Acids Research 43, no. Database issue (2015): D1071–D1078. 10.1093/nar/gku1011 PMC4383880 25348409

[35] D. M. Sanderson and C. G. Earnshaw , “Computer Prediction of Possible Toxic Action From Chemical Structure; the DEREK System,” Human & Experimental Toxicology 10, no. 4 (1991): 261–273. 10.1177/096032719101000405 1679649

[36] A. E. Seiler and H. Spielmann , “The Validated Embryonic Stem Cell Test to Predict Embryotoxicity In Vitro,” Nature Protocols 6, no. 7 (2011): 961–978. 10.1038/nprot.2011.348 21720311

[37] R. A. Shparberg , H. J. Glover , and M. B. Morris , “Embryoid Body Differentiation of Mouse Embryonic Stem Cells Into Neurectoderm and Neural Progenitors,” Methods in Molecular Biology 2029 (2019): 273–285. 10.1007/978-1-4939-9631-5_21 31273749

[38] P. D. Simoes and T. Ramos , “Human Pluripotent Embryonal Carcinoma NTERA2 Cl.D1 Cells Maintain Their Typical Morphology in an Angiomyogenic Medium,” Journal of Negative Results in Biomedicine 6 (2007): 5. 10.1186/1477-5751-6-5 PMC1863432 17442106

[39] N. Yin , X. Yao , Z. Qin , Y. L. Wang , and F. Faiola , “Assessment of Bisphenol A (BPA) Neurotoxicity In Vitro With Mouse Embryonic Stem Cells,” Journal of Environmental Sciences (China) 36 (2015): 181–187. 10.1016/j.jes.2015.06.004 26456621

[40] K. Zeevaert , M. H. Elsafi Mabrouk , W. Wagner , and R. Goetzke , “Cell Mechanics in Embryoid Bodies,” Cells 9, no. 10 (2020): 2270. 10.3390/cells9102270 PMC7599659 33050550

[41] H. Chen , M. A. Chidboy , and J. F. Robinson , “Retinoids and Developmental Neurotoxicity: Utilizing Toxicogenomics to Enhance Adverse Outcome Pathways and Testing Strategies,” Reproductive Toxicology 96 (2020): 102–113. 10.1016/j.reprotox.2020.06.007 PMC7736340 32544423

[42] N. M. Radio and W. R. Mundy , “Developmental Neurotoxicity Testing In Vitro: Models for Assessing Chemical Effects on Neurite Outgrowth,” Neurotoxicology 29, no. 3 (2008): 361–376. 10.1016/j.neuro.2008.02.011 18403021

[43] F. Megiorni , B. Mora , P. Indovina , and M. C. Mazzilli , “Expression of Neuronal Markers During NTera2/cloneD1 Differentiation by Cell Aggregation Method,” Neuroscience Letters 373, no. 2 (2005): 105–109. 10.1016/j.neulet.2004.09.070 15567562

[44] A. K. Menzner , S. Abolpour Mofrad , O. Friedrich , and D. F. Gilbert , “Towards In Vitro DT/DNT Testing: Assaying Chemical Susceptibility in Early Differentiating NT2 Cells,” Toxicology 338 (2015): 69–76. 10.1016/j.tox.2015.10.007 26498558

[45] A. L. van Boxtel , B. Pieterse , P. Cenijn , et al., “Dithiocarbamates Induce Craniofacial Abnormalities and Downregulate sox9a During Zebrafish Development,” Toxicological Sciences 117, no. 1 (2010): 209–217. 10.1093/toxsci/kfq169 20530235

[46] I. R. Edwards , D. G. Ferry , and W. A. Temple , “Fungicides & Related Compounds,” in Handbook of Pesticide Toxicology, vol. 4–2 (Academic Press, 1991).

[47] WHO Fa , “Evaluation of the Toxicity of Pesticide Residues in Food,” (1965).

[48] IARC , “Occupational Exposures in Insecticide Application, and Some Pesticides,” International Agency for Research on Cancer 53 (1991): 1–612.

[49] Z. Wei , S. Sakamuru , L. Zhang , et al., “Identification and Profiling of Environmental Chemicals That Inhibit the TGFbeta/SMAD Signaling Pathway,” Chemical Research in Toxicology 32, no. 12 (2019): 2433–2444. 10.1021/acs.chemrestox.9b00228 PMC7341485 31652400

[50] R. D. Wauchope , T. M. Buttler , A. G. Hornsby , P. W. Augustijn‐Beckers , and J. P. Burt , “The SCS/ARS/CES Pesticide Properties Database for Environmental Decision‐Making,” Reviews of Environmental Contamination and Toxicology 123 (1992): 1–155. 1732992

[51] Z. Maznah , M. Halimah , and B. S. Ismail , “Evaluation of the Persistence and Leaching Behaviour of Thiram Fungicide in Soil, Water and Oil Palm Leaves,” Bulletin of Environmental Contamination and Toxicology 100, no. 5 (2018): 677–682. 10.1007/s00128-018-2312-x 29516138

[52] K. Stadler , X. Li , B. Liu , W. Bao , K. Wang , and H. J. Lehmler , “Systematic Review of Human Biomonitoring Studies of Ethylenethiourea, a Urinary Biomarker for Exposure to Dithiocarbamate Fungicides,” Environmental Pollution 292 (2022): 118419. 10.1016/j.envpol.2021.118419 PMC8627121 34751155

[53] D. E. Hines , S. Bell , X. Chang , K. Mansouri , D. Allen , and N. Kleinstreuer , “Application of an Accessible Interface for Pharmacokinetic Modeling and In Vitro to In Vivo Extrapolation,” Frontiers in Pharmacology 13 (2022): 864742. 10.3389/fphar.2022.864742 PMC9043603 35496281

[54] M. Ni , D. Deepika , X. Li , et al., “IVIVE‐PBPK Based New Approach Methodology for Addressing Early Life Toxicity Induced by Bisphenol A,” Environmental Research 240, no. Pt 1 (2024): 117343. 10.1016/j.envres.2023.117343 37858691

[55] X. Chang , J. Palmer , A. Lumen , et al., “Quantitative In Vitro to In Vivo Extrapolation for Developmental Toxicity Potency of Valproic Acid Analogues,” Birth Defects Research 114, no. 16 (2022): 1037–1055. 10.1002/bdr2.2019 PMC9790683 35532929

[56] X. Chang , Y. M. Tan , D. G. Allen , et al., “IVIVE: Facilitating the Use of In Vitro Toxicity Data in Risk Assessment and Decision Making,” Toxics 10, no. 5 (2022): 232. 10.3390/toxics10050232 PMC9143724 35622645

[57] NTP , Integrated Chemical Environment (ICE) PBPK Tool User Guide (Version 4.2) (National Toxicology Program, National Institute of Environmental Health Sciences, 2024), https://ice.ntp.niehs.nih.gov/downloads/UserGuides/PBPK_userguide.pdf.

[58] B. C. Jaleh Abedinia , S. Bella , X. Changa , et al., “Application of New Approach Methodologies: ICE Tools to Support Chemical Evaluations,” Computational Toxicology 20 (2021): 100184.

[59] NHMRC , Thiram | Australian Drinking Water Guidelines (National Health and Medical Research Council, 2025), https://guidelines.nhmrc.gov.au/australian‐drinking‐water‐guidelines/part‐5/physical‐chemical‐characteristics/thiram.

[60] X. Huang and J. P. Saint‐Jeannet , “Induction of the Neural Crest and the Opportunities of Life on the Edge,” Developmental Biology 275, no. 1 (2004): 1–11. 10.1016/j.ydbio.2004.07.033 15464568

[61] O. Shakhova and L. Sommer , Neural Crest‐Derived Stem Cells (StemBook, 2010). 20614636

[62] M. Rocha , N. Singh , K. Ahsan , A. Beiriger , and V. E. Prince , “Neural Crest Development: Insights From the Zebrafish,” Developmental Dynamics 249, no. 1 (2020): 88–111. 10.1002/dvdy.122 PMC7273345 31591788

[63] D. Meulemans and M. Bronner‐Fraser , “Gene‐Regulatory Interactions in Neural Crest Evolution and Development,” Developmental Cell 7, no. 3 (2004): 291–299. 10.1016/j.devcel.2004.08.007 15363405

[64] H. J. Parker , I. Pushel , and R. Krumlauf , “Coupling the Roles of Hox Genes to Regulatory Networks Patterning Cranial Neural Crest,” Developmental Biology 444, no. Suppl 1 (2018): S67–S78. 10.1016/j.ydbio.2018.03.016 29571614

[65] H. J. Parker and R. Krumlauf , “A Hox Gene Regulatory Network for Hindbrain Segmentation,” Current Topics in Developmental Biology 139 (2020): 169–203. 10.1016/bs.ctdb.2020.03.001 32450960

[66] T. H. Vu , M. Takechi , M. Shimizu , et al., “Dlx5‐Augmentation in Neural Crest Cells Reveals Early Development and Differentiation Potential of Mouse Apical Head Mesenchyme,” Scientific Reports 11, no. 1 (2021): 2092. 10.1038/s41598-021-81434-x PMC7822927 33483579

[67] M. Shimizu , N. Narboux‐Neme , Y. Gitton , et al., “Probing the Origin of Matching Functional Jaws: Roles of Dlx5/6 in Cranial Neural Crest Cells,” Scientific Reports 8, no. 1 (2018): 14975. 10.1038/s41598-018-33207-2 PMC6175850 30297736

[68] B. A. Firulli , R. K. Fuchs , J. W. Vincentz , D. E. Clouthier , and A. B. Firulli , “Hand1 Phosphoregulation Within the Distal Arch Neural Crest Is Essential for Craniofacial Morphogenesis,” Development 141, no. 15 (2014): 3050–3061. 10.1242/dev.107680 PMC4197675 25053435

[69] H. E. Seberg , E. Van Otterloo , S. K. Loftus , et al., “TFAP2 Paralogs Regulate Melanocyte Differentiation in Parallel With MITF,” PLoS Genetics 13, no. 3 (2017): e1006636. 10.1371/journal.pgen.1006636 PMC5352137 28249010

[70] E. Van Otterloo , W. Li , G. Bonde , K. M. Day , M. Y. Hsu , and R. A. Cornell , “Differentiation of Zebrafish Melanophores Depends on Transcription Factors AP2 Alpha and AP2 Epsilon,” PLoS Genetics 6, no. 9 (2010): e1001122. 10.1371/journal.pgen.1001122 PMC2940735 20862309

[71] B. M. K. Vasamsetti , K. Chon , J. Y. Choi , J. Kim , and C. Y. Yoon , “Transcriptome Analysis of Thiram‐Treated Zebrafish ( *Danio rerio* ) Embryos Reveals Disruption of Reproduction Signaling Pathways,” Biology‐Basel 12, no. 2 (2023): 156. 10.3390/biology12020156 PMC9953208 36829436

[72] Y. X. Li , M. Zdanowicz , L. Young , D. Kumiski , L. Leatherbury , and M. L. Kirby , “Cardiac Neural Crest in Zebrafish Embryos Contributes to Myocardial Cell Lineage and Early Heart Function,” Developmental Dynamics 226, no. 3 (2003): 540–550. 10.1002/dvdy.10264 12619138

[73] M. R. Hutson and M. L. Kirby , “Model Systems for the Study of Heart Development and Disease. Cardiac Neural Crest and Conotruncal Malformations,” Seminars in Cell & Developmental Biology 18, no. 1 (2007): 101–110. 10.1016/j.semcdb.2006.12.004 PMC1858673 17224285

[74] H. C. Etchevers , C. Vincent , N. M. Le Douarin , and G. F. Couly , “The Cephalic Neural Crest Provides Pericytes and Smooth Muscle Cells to All Blood Vessels of the Face and Forebrain,” Development 128, no. 7 (2001): 1059–1068. 10.1242/dev.128.7.1059 11245571

[75] M. R. Hutson and M. L. Kirby , “Neural Crest and Cardiovascular Development: A 20‐Year Perspective,” Birth Defects Research. Part C, Embryo Today: Reviews 69, no. 1 (2003): 2–13. 10.1002/bdrc.10002 12768653

[76] A. Keyte and M. R. Hutson , “The Neural Crest in Cardiac Congenital Anomalies,” Differentiation 84, no. 1 (2012): 25–40. 10.1016/j.diff.2012.04.005 PMC3389200 22595346

[77] A. K. Bal‐Price , H. T. Hogberg , L. Buzanska , and S. Coecke , “Relevance of In Vitro Neurotoxicity Testing for Regulatory Requirements: Challenges To Be Considered,” Neurotoxicology and Teratology 32, no. 1 (2010): 36–41. 10.1016/j.ntt.2008.12.003 19150401

[78] A. K. Bal‐Price , H. T. Hogberg , L. Buzanska , P. Lenas , E. van Vliet , and T. Hartung , “In Vitro Developmental Neurotoxicity (DNT) Testing: Relevant Models and Endpoints,” Neurotoxicology 31, no. 5 (2010): 545–554. 10.1016/j.neuro.2009.11.006 19969020

[79] J. P. Frimat , J. Sisnaiske , S. Subbiah , et al., “The Network Formation Assay: A Spatially Standardized Neurite Outgrowth Analytical Display for Neurotoxicity Screening,” Lab on a Chip 10, no. 6 (2010): 701–709. 10.1039/b922193j 20221557

[80] C. van Thriel , R. H. Westerink , C. Beste , A. S. Bale , P. J. Lein , and M. Leist , “Translating Neurobehavioural Endpoints of Developmental Neurotoxicity Tests Into In Vitro Assays and Readouts,” Neurotoxicology 33, no. 4 (2012): 911–924. 10.1016/j.neuro.2011.10.002 PMC3288589 22008243

[81] D. H. Baek , T. G. Kim , H. K. Lim , et al., “Embryotoxicity Assessment of Developmental Neurotoxicants Using a Neuronal Endpoint in the Embryonic Stem Cell Test,” Journal of Applied Toxicology 32, no. 8 (2012): 617–626. 10.1002/jat.1747 22131109

