# Supplementary material for: Evaluating Thiram‐Induced Embryotoxicity Using Integrated In Silico, In Vitro, and Transcriptomic Approaches
Source: Environ Toxicol. 2026 Apr 21;41(12):1798–812. doi: 10.1002/tox.70103 (PMC13749236; doi:10.1002/tox.70103)
Supplement: Supplementary file 1 [file TOX-41-1798-s001.docx]

**Table S1. The RT-PCR was performed with the following TaqMan™ Assay**

| **Gene names** | **Assay ID** |
| --- | --- |
| **Mouse** |  |
| *Oct4* | Mm03053917_g1 |
| *Sox2* | Mm0305810_s1 |
| *Nanog* | Mm02019550_s1 |
| *Fgf5* | Mm00438919_m1 |
| *Brachyury T* | Mm00436877_m1 |
| *Gata6* | Mm00802636_m1 |
| *Sox1* | Mm00486299_s1 |
| *Penk1* | Mm01212875_m1 |
| *Hoxa1* | Mm00439359_m1 |
| *Nestin* | Mm00450205_m1 |
| *NeuN* | Mm01248781_m1 |
| *Gapdh* | Mm99999915_g1 |
| **Human** |  |
| *OCT4* | Hs00999632_g1 |
| *SOX2* | Hs04234836_s1 |
| *NANOG* | Hs02387400_g1 |
| *NESTIN* | Hs04187831_g1 |
| *NEUN* | Hs01370654_m1 |
| *MAP2* | Hs00258900_m1 |
| *HOXA1* | Hs00939046_m10 |
| *GAPDH* | Hs99999905_m1 |

**Table S2. The RT-PCR was performed with the following SYBR Green primers**

| **Gene names** | **SYBR Green primers** |
| --- | --- |
| *DLX5 F* | AGCCATGTCTGCTTAGACCA |
| *DLX5 R* | AACACTCCTGTCATCGCTCA |
|  |  |
| *HOXB1 F* | CCCTGCCCTTCAGAACCTAA |
| *HOXB1 R* | CTGACACCTTCGCTGTCTTG |
|  |  |
| *TFAP2A F* | TTCCTGCCAAAGCAGTAGC |
| *TFAP2A R* | CAGGTCGGTGAACTCTTTGC |
|  |  |
| *BMP4 F* | CGGAAGCTAGGTGAGTGTGG |
| *BMP4 R* | TCGAGATAGCTTGGACGGGA |
|  |  |
| *ISL1 F* | TCTTTCAGCATTGGCAACCC |
| *ISL1 R* | GAACAGCCCACAGAGAGGAT |
|  |  |
| *HAND1 F* | AAAGGCTCAGGACCCAAGAA |
| *HAND1 R* | TCAGGTAGGCGATGTAGCTG |

**Table S3. The WoE scores of 127 FCMs**

| **CAS** | **Chemical name** | **Source** | **Predict score** | **TTC** |
| --- | --- | --- | --- | --- |
| 67-56-1 | Methanol | Swiss printing ink, EU | 0.90 | Low (Class I) |
| **2321-07-5** | **Solvent Yellow 94**  **(Fluorescein)** | **Swiss printing ink** | **0.89** | **High (Class III)** |
| 56-23-5 | CARBON TETRACHLORIDE | US FDA+Karmaus et al | 0.88 | High (Class III) |
| 75-12-7 | Formamide | US FDA+Karmaus et al,  Swiss printing ink | 0.85 | High (Class III) |
| 25014-41-9 | POLYACRYLONITRILE | US FDA+Karmaus et al | 0.84 | High (Class III) |
| 107-13-1 | Acrylonitrile | US FDA+Karmaus et al, Swiss printing ink, EU | 0.84 | High (Class III) |
| 9002-86-2 | Polyvinyl chloride | US FDA+Karmaus et al,  Swiss printing ink | 0.83 | High (Class III) |
| 75-01-4 | Vinyl Chloride | US FDA+Karmaus et al,  Swiss printing ink, EU | 0.83 | High (Class III) |
| 75-15-0 | CARBON DISULFIDE | US FDA+Karmaus et al | 0.83 | High (Class III) |
| 107-12-0 | Propionitrile | Swiss printing ink | 0.81 | High (Class III) |
| 540-59-0 | 1,2-DICHLOROETHYLENE | US FDA+Karmaus et al | 0.80 | High (Class III) |
| 106-89-8 | Epichlorohydrin | US FDA+Karmaus et al,  Swiss printing ink, EU | 0.80 | High (Class III) |
| 74-87-3 | Methane, chloro- | Swiss printing ink | 0.80 | High (Class III) |
| 63022-06-0 | Pigment Red 81:5 | Swiss printing ink | 0.80 | High (Class III) |
| 62-56-6 | Urea, thio- | Swiss printing ink | 0.80 | High (Class III) |
| 75-00-3 | Ethyl chloride | Swiss printing ink | 0.79 | High (Class III) |
| 3033-77-0 | Ammonium, (2,3-epoxypropyl)trimethyl-, chloride | US FDA+Karmaus et al, Swiss printing ink | 0.79 | High (Class III) |
| 81-48-1 | Solvent Violet 13 | Swiss printing ink | 0.78 | High (Class III) |
| 75-21-8 | Ethylene oxide | Swiss printing ink, EU | 0.78 | High (Class III) |
| 556-52-5 | 2,3-Epoxypropanol | Swiss printing ink | 0.77 | High (Class III) |
| 57-13-6 | Urea | Swiss printing ink, EU | 0.76 | High (Class III) |
| 9002-85-1 | POLYVINYLIDENE CHLORIDE | US FDA+Karmaus et al | 0.75 | High (Class III) |
| 75-35-4 | Vinylidene chloride | US FDA+Karmaus et al, Swiss printing ink, EU | 0.75 | High (Class III) |
| **15086-94-9** | **Solvent Red 43(Eosin Y)** | **Swiss printing ink** | **0.75** | **High (Class III)** |
| 6359-98-4 | Acid Yellow 17 | Swiss printing ink | 0.75 | Low (Class I) |
| 6471-50-7 | Pigment Red 14 | Swiss printing ink | 0.73 | High (Class III) |
| **CAS** | **Chemical name** | **Source** | **Predict score** | **TTC** |
| 67969-87-3 | Direct Yellow 118 | Swiss printing ink | 0.73 | High (Class III) |
| 68516-75-6 | Pigment Brown 41 | Swiss printing ink | 0.73 | High (Class III) |
| 72705-26-1 | Direct Yellow 157 | Swiss printing ink | 0.73 | Low (Class I) |
| 85455-55-6 | 3H-Indolium, 2-[2-[4-[(2-ethoxyethyl)ethylamino]-2-methyl- phenyl]ethenyl]-1,3,3-trimethyl-, (T-4)-tetrachlorozincate(2-) (2:1) | Swiss printing ink | 0.73 | High (Class III) |
| 2478-21-9 | 2,6-BIS((DIMETHYLAMINO)METHYL)CYCLOHEXANONE | US FDA+Karmaus et al | 0.73 | High (Class III) |
| 26638-19-7 | DICHLOROPROPANE | US FDA+Karmaus et al | 0.73 | High (Class III) |
| 30105-01-2 | DIMETHYL-ALPHA-METHYLSTYRENE | US FDA+Karmaus et al | 0.73 | Low (Class I) |
| 91815-41-7 | TETRAHYDRO-4-HYDROXY-5-METHYL-2(1H)PYRIMIDINONE | US FDA+Karmaus et al | 0.73 | High (Class III) |
| 1328-53-6 | Pigment Green 7 | US FDA+Karmaus et al, Swiss printing ink | 0.73 | High (Class III) |
| 14302-13-7 | Pigment Green 36 | Swiss printing ink | 0.73 | High (Class III) |
| 65212-77-3 | 4,5-DICHLORO-2-((5-HYDROXY-3-METHYL-1-(3-SULFOPHENYL)-1H-PYRAZOL-4-YL)AZO)BENZENESULFONIC ACID, CALCIUM SALT(1:1) | US FDA+Karmaus et al, PIDC | 0.73 | Low (Class I) |
| 22094-93-5 | Pigment Yellow 81 | Swiss printing ink | 0.73 | High (Class III) |
| 13003-12-8 | 4,4'-Butylidene-bis(6-tert-butyl-3-methylphenyl-ditridecyl phosphite) | Swiss printing ink, EU | 0.73 | High (Class III) |
| 3905-19-9 | Pigment Red 166 | Swiss printing ink | 0.73 | High (Class III) |
| 71566-54-6 | Pigment Red 221 | Swiss printing ink | 0.73 | High (Class III) |
| 5280-78-4 | Pigment Red 144 | Swiss printing ink | 0.73 | High (Class III) |
| 68937-66-6 | Alcohols, C6-12, ethoxylated propoxylated | Swiss printing ink | 0.73 | High (Class III) |
| **CAS** | **Chemical name** | **Source** | **Predict score** | **TTC** |
| 40618-31-3 | Pigment Red 214 | Swiss printing ink | 0.73 | High (Class III) |
| 61847-48-1 | Pigment Red 188 | Swiss printing ink | 0.73 | High (Class III) |
| 52238-92-3 | Pigment Red 242 | Swiss printing ink | 0.73 | High (Class III) |
| 90268-24-9 | Pigment Yellow 176 | Swiss printing ink | 0.73 | High (Class III) |
| 12239-15-5 | Acid Yellow 49 | Swiss printing ink | 0.73 | High (Class III) |
| 73612-29-0 | 2-Naphthalenecarboxylic acid, 3-hydroxy-4-[(4-methyl-2-sul- fophenyl)azo]-, strontium salt (1:1) | Swiss printing ink | 0.73 | High (Class III) |
| 35869-64-8 | Pigment Brown 23 | Swiss printing ink | 0.73 | High (Class III) |
| 3214-47-9 | Direct Yellow 50 | Swiss printing ink | 0.73 | High (Class III) |
| 56797-01-4 | Hexanoic acid, 2-ethyl-, cerium(3+) salt | Swiss printing ink | 0.73 | High (Class III) |
| 68259-05-2 | Pigment Red 220 | US FDA+Karmaus et al, Swiss printing ink, PIDC | 0.73 | High (Class III) |
| 76233-81-3 | Pigment Violet 50 | Swiss printing ink | 0.73 | High (Class III) |
| 89959-98-8 | 1H-1,2,4-Triazolium, 1,2(or 1,4)-dimethyl-3(or5)-[[4-[methyl (phenylmethyl) amino]phenyl]azo]-, bromide | Swiss printing ink | 0.73 | High (Class III) |
| 20018-09-1 | 4-(DIIODOMETHYLSULFONYL)TOLUENE | US FDA+Karmaus et al | 0.73 | High (Class III) |
| 5979-28-2 | Pigment Yellow 16 | Swiss printing ink | 0.73 | High (Class III) |
| 6245-59-6 | Acid Red 6 | Swiss printing ink | 0.73 | High (Class III) |
| 6505-30-2 | Acid Blue 104 | Swiss printing ink | 0.73 | High (Class III) |
| 10228-01-0 | Quino[2,3-b]acridine-7,14-dione, 5,12-dihydro-2-methyl- | Swiss printing ink | 0.73 | High (Class III) |
| 24593-34-8 | Cerium-2-ethylhexanoate | US FDA+Karmaus et al, Swiss printing ink | 0.73 | High (Class III) |
| 38720-66-0 | Pigment Red 209 | Swiss printing ink | 0.73 | High (Class III) |
| 50662-99-2 | Reactive Yellow 2 | Swiss printing ink | 0.73 | Low (Class I) |
| **CAS** | **Chemical name** | **Source** | **Predict score** | **TTC** |
| 152165-12-3 | Perylene-3,4-dicarboximide-9-sulphonic acid | Swiss printing ink | 0.73 | High (Class III) |
| 77-40-7 | BISPHENOL B | US FDA+Karmaus et al | 0.72 | High (Class III) |
| 97-77-8 | DISULFIRAM | US FDA+Karmaus et al | 0.68 | High (Class III) |
| 64-17-5 | Ethanol | Swiss printing ink, EU | 0.66 | Low (Class I) |
| 80-05-7 | 2,2-Bis(4-hydroxyphenyl)propane | US FDA+Karmaus et al, Swiss printing ink, EU | 0.65 | High (Class III) |
| 9000-14-0 | Copals | Swiss printing ink | 0.64 | High (Class III) |
| **137-26-8** | **THIRAM** | **US FDA+Karmaus et al** | **0.62** | **High (Class III)** |
| 93-35-6 | UMBELLIFERONE | US FDA+Karmaus et al | 0.60 | High (Class III) |
| 117-81-7 | Phthalic acid, bis(2-ethylhexyl) ester | US FDA+Karmaus et al, Swiss printing ink, EU | 0.60 | Low (Class I) |
| 85-60-9 | 1,1-Bis(2-methyl-4-hydroxy-5-tert-butylphenyl)butane | US FDA+Karmaus et al, Swiss printing ink | 0.59 | High (Class III) |
| **119-47-1** | **2,2'-Methylenebis(4-methyl-6-tert-butylphenol)** | **US FDA+Karmaus et al, Swiss printing ink, EU** | **0.59** | **High (Class III)** |
| 140-66-9 | 4-(1,1,3,3-Tetramethylbutyl)phenol | Swiss printing ink | 0.58 | Low (Class I) |
| 3734-33-6 | Ammonium, benzyldiethyl[(2,6-xylylcarbamoyl)methyl]-, benzoate | Swiss printing ink | 0.56 | High (Class III) |
| 1817-68-1 | 2,6-DI(ALPHA-METHYL BENZYL)-4-METHYL PHENOL | US FDA+Karmaus et al | 0.55 | High (Class III) |
| 128-04-1 | Carbamic acid, dimethyldithio-, sodium salt | Swiss printing ink | 0.55 | High (Class III) |
| 1260-17-9 | Natural Red 4 | Swiss printing ink | 0.53 | Intermediate (Class II) |
| 124-38-9 | Carbon dioxide | Swiss printing ink, EU | 0.53 | High (Class III) |
| 7782-44-7 | OXYGEN | US FDA+Karmaus et al | 0.53 | High (Class III) |
| 79-94-7 | 2,2-Bis(3,5-dibromo-4-hydroxyphenyl)propane | Swiss printing ink | 0.53 | High (Class III) |
| 7732-18-5 | Water | EU | 0.52 | High (Class III) |
| **CAS** | **Chemical name** | **Source** | **Predict score** | **TTC** |
| 7440-23-5 | SODIUM | US FDA+Karmaus et al | 0.52 | High (Class III) |
| 85305-20-0 | Phenol, 2,4,6-tris[1-(methylphenyl)ethyl]- | Swiss printing ink | 0.52 | High (Class III) |
| 1620-68-4 | 2ALPHA,6ALPHA-BIS(6-HYDROXY-M-TOLYL)MESITOL | US FDA+Karmaus et al | 0.52 | High (Class III) |
| 7782-41-4 | FLUORINE | US FDA+Karmaus et al | 0.52 | High (Class III) |
| 7440-66-6 | Pigment Black 16 | US FDA+Karmaus et al, Swiss printing ink | 0.52 | High (Class III) |
| 7779-90-0 | ZINC ORTHOPHOSPHATE | US FDA+Karmaus et al | 0.52 | High (Class III) |
| 7722-84-1 | Hydrogen peroxide | Swiss printing ink | 0.52 | High (Class III) |
| 8007-30-5 | HYDROGEN PEROXIDE SOLUTION | US FDA+Karmaus et al | 0.52 | High (Class III) |
| 10043-52-4 | Calcium chloride | Swiss printing ink, EU | 0.52 | High (Class III) |
| 7704-34-9 | Sulphur | US FDA+Karmaus et al, Swiss printing ink, EU | 0.52 | High (Class III) |
| 1310-73-2 | Sodium hydroxide | Swiss printing ink, EU | 0.52 | High (Class III) |
| 14066-20-7 | DIHYDROGEN PHOSPHATE | US FDA+Karmaus et al | 0.52 | High (Class III) |
| 14066-19-4 | HYDROGEN PHOSPHATE | US FDA+Karmaus et al | 0.52 | High (Class III) |
| 7664-38-2 | Phosphoric acid | Swiss printing ink, EU | 0.52 | High (Class III) |
| 7439-93-2 | LITHIUM | US FDA+Karmaus et al | 0.52 | High (Class III) |
| 3097-08-3 | MAGNESIUM LAURYL SULFATE | US FDA+Karmaus et al | 0.52 | High (Class III) |
| 7631-86-9 | Silicon dioxide | Swiss printing ink, EU | 0.52 | High (Class III) |
| 14808-60-7 | Quartz | Swiss printing ink, EU | 0.52 | High (Class III) |
| 60676-86-0 | Silica, vitreous (coatings) | Swiss printing ink | 0.52 | High (Class III) |
| 68909-20-6 | Silanamine, 1,1,1-trimethyl-N-(trimethylsilyl)-, hydrolysis products with silica | Swiss printing ink | 0.52 | High (Class III) |
| 14464-46-1 | Cristobalite | Swiss printing ink, EU | 0.52 | High (Class III) |
| 61790-53-2 | Diatomaceous earth | Swiss printing ink, EU | 0.52 | High (Class III) |
| 7757-82-6 | SODIUM SULFATE, ANHYDROUS | US FDA+Karmaus et al | 0.52 | High (Class III) |
| **CAS** | **Chemical name** | **Source** | **Predict score** | **TTC** |
| 7664-93-9 | Sulphuric acid | Swiss printing ink, EU | 0.52 | High (Class III) |
| 10039-54-0 | Hydroxylamine, sulfate (2:1) (salt) | US FDA+Karmaus et al, Swiss printing ink | 0.52 | High (Class III) |
| 15181-46-1 | BISULFITE | US FDA+Karmaus et al | 0.51 | High (Class III) |
| 7647-01-0 | Hydrochloric acid | Swiss printing ink, EU | 0.51 | High (Class III) |
| 94-26-8 | Benzoic acid, p-hydroxy-, butyl ester | Swiss printing ink | 0.51 | Low (Class I) |
| 7440-09-7 | POTASSIUM | US FDA+Karmaus et al | 0.51 | High (Class III) |
| 7705-08-0 | Iron chloride, (FeCl3) | Swiss printing ink | 0.51 | High (Class III) |
| 10034-85-2 | HYDRIODIC ACID | US FDA+Karmaus et al | 0.51 | High (Class III) |
| 7681-82-5 | Sodium iodide | US FDA+Karmaus et al, Swiss printing ink, EU | 0.51 | High (Class III) |
| 7439-96-5 | MANGANESE | US FDA+Karmaus et al | 0.51 | High (Class III) |
| 7697-37-2 | Nitric acid | US FDA+Karmaus et al, Swiss printing ink | 0.50 | High (Class III) |
| 6484-52-2 | AMMONIUM NITRATE | US FDA+Karmaus et al | 0.50 | High (Class III) |
| 7601-54-9 | Sodium phosphate tribasic | Swiss printing ink | 0.50 | High (Class III) |
| 13462-86-7 | Barite | Swiss printing ink | 0.50 | High (Class III) |
| 7727-43-7 | Pigment White 21 | US FDA+Karmaus et al, Swiss printing ink | 0.50 | High (Class III) |
| 94-13-3 | 4-Hydroxybenzoic acid, propyl ester | Swiss printing ink, EU | 0.50 | Low (Class I) |
| 35285-69-9 | Benzoic acid, 4-hydroxy-, propyl ester, sodium salt | Swiss printing ink | 0.50 | High (Class III) |

**B.**

**A.**

**D.**

**C.**

**E.**

**Figure S1. Effect of thiram on expression of differentiation-related genes in mESCs.**

The mESCs were treated with DMSO or 0.15 μg/ml thiram for 3-10 days in mESCs-derived cardiomyocyte differentiation. Differentiation markers included genes for (A) Oct4, (B) Gata6, (C) Fgf5 (D) Brachyury T and (E) Tnnt2. The results were presented as mean ± standard deviation (SD) of one independent experiment with 3 replicates in each experiment. *p < 0.05, compared with DMSO-treated cells.
